# Identification of SNP loci and candidate genes genetically controlling norisoprenoids in grape berry based on genome-wide association study

**DOI:** 10.3389/fpls.2023.1142139

**Published:** 2023-03-01

**Authors:** Qi Sun, Lei He, Lei Sun, Hai-Ying Xu, Ya-Qun Fu, Zheng-Yang Sun, Bao-Qing Zhu, Chang-Qing Duan, Qiu-Hong Pan

**Affiliations:** ^1^ Center for Viticulture & Enology, College of Food Science and Nutritional Engineering, China Agricultural University, Beijing, China; ^2^ Key Laboratory of Viticulture and Enology, Ministry of Agriculture and Rural Affairs, Beijing, China; ^3^ Beijing Academy of Forestry and Pomology Sciences, Beijing, China; ^4^ College of Biological Sciences and Biotechnology, Beijing Forestry University, Beijing, China

**Keywords:** grape berry, norisoprenoids, single nucleotide polymorphism, genome-wide association study, genetic regulation

## Abstract

Obtaining new grapevine varieties with unique aromas has been a long-standing goal of breeders. Norisoprenoids are of particular interest to wine producers and researchers, as these compounds are responsible for the important varietal aromas in wine, characterized by a complex floral and fruity smell, and are likely present in all grape varieties. However, the single-nucleotide polymorphism (SNP) loci and candidate genes genetically controlling the norisoprenoid content in grape berry remain unknown. To this end, in this study, we investigated 13 norisoprenoid traits across two years in an F_1_ population consisting of 149 individuals from a hybrid of *Vitis vinifera* L. cv. Muscat Alexandria and *V. vinifera* L. cv. Christmas Rose. Based on 568,953 SNP markers, genome-wide association analysis revealed that 27 candidate SNP loci belonging to 18 genes were significantly associated with the concentrations of norisoprenoid components in grape berry. Among them, 13 SNPs were confirmed in a grapevine germplasm population comprising 97 varieties, including two non-synonymous mutations SNPs within the *VvDXS1* and *VvGGPPS* genes, respectively in the isoprenoid metabolic pathway. Genotype analysis showed that the grapevine individuals with the heterozygous genotype C/T at chr5:2987350 of *VvGGPPS* accumulated higher average levels of 6-methyl-5-hepten-2-one and *β*-cyclocitral than those with the homozygous genotype C/C. Furthermore, *VvGGPPS* was highly expressed in individuals with high norisoprenoids concentrations. Transient overexpression of *VvGGPPS* in the leaves of *Vitis quinquangularis* and tobacco resulted in an increase in norisoprenoid concentrations. These findings indicate the importance of *VvGGPPS* in the genetic control of norisoprenoids in grape berries, serving as a potential molecular breeding target for aroma.

## 1 Introduction

Grapes (*Vitis* spp.) are the most economically important fruit species in the world. It is estimated that the global vineyard area is approximately 6950 kilo-hectares, with an yield of 78,034 kilo-tonnes in 2020, ranking first in fruit production (From FAO data). Approximately 71% of this production is used for wine, 27% for fresh fruit, and 2% for raisins. More than 50 species in the *Vitis* genus have been recognized to date. *Vitis* germplasm constitutes a valuable resource for obtaining desired traits, such as increased tolerance to pathogens and improved flavor quality, through breeding programs. New grapevine varieties with unique flavors are expected to be bred to meet diverse consumption needs. During the last several decades, grape breeding worldwide has aimed at developing new varieties with disease resistance, unique flavors and better quality (Powell, 2008; Reisch et al., 2012). Aroma breeding has also long attracted great attention, as aroma characteristics are important indicators for both fresh fruit consumption and processing. The most important aromatic compounds in grape berries are monoterpenoids and norisoprenoids, which are generated *via* isoprenoid metabolism.

Monoterpenoids are primarily important in the Muscat-type and non-Muscat aromatic-type grape varieties, whereas norisoprenoids, with very low odor perception thresholds, are the main contributors to the floral and fruity scents in the neutral grape varieties and the wines produced from these grapes. (Strauss et al., 1987; Mateo and Jimenez, 2000; Kwasniewski et al., 2010; Robinson et al., 2014; Gao et al., 2016; Yuan and Qian, 2016; Zhang et al., 2016). Norisoprenoids are produced by the cleavage of a group of carotenoids with 40 carbons (C40) in the plastids. Carotenoids are derived from the sequential condensation of 5-carbon (C5) building blocks, namely isopentenyl diphosphate (IPP), and its double-bond isomer dimethylallyl diphosphate (DMAPP). The biosynthesis of these two universal precursors in plant cells involves two independent pathways: the mevalonic acid (MVA) pathway in the cytosol and the methyl-erythritol-phosphate (MEP) pathway in the plastids. Most monoterpenes (C10), diterpenes (C20), and tetraterpenes (C40), such as carotenoids, are derive from the MEP pathway, with 1-deoxy-D -xylulose-5-phosphate synthase (DXS) as the entrance and rate-limiting enzyme (Winterhalter and Rouseff, 2002; Schwab et al., 2008; Dunlevy et al., 2009; May et al., 2013; Schwab and Wust, 2015). In plastids, geranylgeranyl diphosphate synthase (GGPPS) acts at the intersection, leading to the C10 monoterpene synthetic branch and the C20/C40 compounds synthetic branch. Thereafter, carotenoids are oxidatively cleaved by carotenoid cleavage dioxygenases (CCDs) or by non-enzymatic reactions into norisoprenoids derivatives with C9, C10, C11, and C13 (Mendes-Pinto, 2009). In grape berries, there are three CCDs that have been biochemically identified, being VvCCD1, VvCCD4a, and VvCCD4b. All three enzymes can cleave lycopene at the 5, 6 (5’, 6’) position to generate 6-methyl-5-hepten-2-one (MHO). VvCCD1 cleaves *β*-carotene at the 9, 10 (9’, 10’) position to form *β*-ionone, while VvCCD4b cleaves *ξ*-carotene at the 9, 10 (9’, 10’) position to form geranylacetone (Lashbrooke et al., 2013). In *Crocus sativus*, CsCCD4c cleaves *β*-carotene at different sites to produce *β*-ionone and *β*-cyclocitral (Rubio-Moraga et al., 2014).

Currently, the norisoprenoid derivatives that have been chemically identified in grape berries and wine include *β*-damascenone (C13), *β*-ionone (C13), 1,1,5-trimethyl-1,2-dihydronapthalene (TDN; C13), *β*-cyclocitral (C10), geranylacetone (C13), 2,2,6-trimethylcyclohexanone (TCH; C9), (E)-1-(2,3,6-trimethylphenyl) buta-1,3-diene (TPB; C13), theaspirane (C13), vitispirane (C13), riesling acetal (C13), and MHO (C8) (Mendes-Pinto, 2009). From the perspective of aroma description, *β*-damascenone mainly presents a “floral”, “honey”, “cooked apple”, or tropical fruit scent in wine, with a sensory threshold of only 50 ng/L in model wine (Kotseridi, 1999). *β*-Ionone exhibits “violet” and “raspberry” aromas, with a sensory threshold of 90 ng/L in model wine (Aznar et al., 2001). TDN, with a sensory threshold in wine of 20 μg/L, exhibits “kerosene” or “gasoline” notes and is considered a characteristic aroma compound of *V. vinifera* riesling wines (Simpson, 1978). TCH mainly exhibits a “rosy” aroma with a sensory threshold of 44.3 μg/L in water (Silva Ferreira and Guedes de Pinho, 2004). Vitispirane mainly presents “eucalyptus” or “camphor” aromas (Genovese et al., 2007). Other compounds such as riesling acetal, MHO, geranylacetone, and *β*-cyclocitral are characterized by “lemon”, “citrus”, and other tropical fruit scents (Zhang et al., 2017). Importantly, obtaining grape varieties with abundant aroma compounds, such as norisoprenoid derivatives, will be of significance in enriching the consumption of fresh fruit and wines.

Aroma compound accumulation in fruits is affected by a variety of factors, including genetic background, growth environment, and cultivation practices (Jackson and Lombard, 1993; Ghaste et al., 2015). At the genetic level, aroma is a complex quantitative trait controlled by multiple genes. With rapid developments in sequencing and detection technologies, genealogy-based linkage mapping and natural population-based association analysis are currently the main methods for conducting quantitative trait studies in grapevines and other fruit crops. Many quantitative traits have been genetically dissected in grapevines, such as berry weight and number of fruit seeds (Doligez et al., 2002; Doligez et al., 2013), berry hardness (Carreño et al., 2014; Jiang et al., 2020), berry sugar and acid (Chen et al., 2015; Bayo-Canha et al., 2019), phenological stage (Duchene et al., 2009), anthocyanin (Ban et al., 2014; Fournier-Level et al., 2014), flavonoids (Malacarne et al., 2015), downy mildew -resistance (Sargolzaei et al., 2020), and powdery mildew -resistance (Blanc et al., 2012). Regarding the aroma traits of grape berry, previous studies have primarily focused on terpenes and methoxypyrazines. First, the quantitative trait loci (QTLs) related to the levels of linalool, nerol and geraniol, the three main monoterpenes in grape berry, were identified on linkage groups (LGs) 1, 5, and 7, and the QTLs with major effects explaining 17–55% of the total phenotypic variance were found to be localized on LG5. Meanwhile, two QTLs controlling the linalool content were mapped on LG10 (Doligez et al., 2006). Next, the gene encoding VvDXS, the first enzyme in the plastidial isoprenoid biosynthesis pathway, was found to co-localize with a major QTL for the levels of linalool, nerol and geraniol on LG 5 in 2009 (Battilana et al., 2009). Two years later, a single-nucleotide polymorphism (SNP) locus (named SNP1822) within *VvDXS* was confirmed, causing the substitution of lysine with an asparagine at position 284 in VvDXS, and this non-synonymous mutation increased the catalytic efficiency by altering the enzyme kinetics (Battilana et al., 2011). *VvDXS* has been proven to genetically control the monoterpene content in grape berries. A recent genome-wide association study (GWAS) identified three QTLs located close to the functional genes *DXS*, *FPPS*, and *HDR* associated with the linalool and geraniol aroma compounds from 96 grapevine cultivars, however, no further verification experiment has been conducted (Yang et al., 2017).

Methoxypyrazines (MPs), a class of compounds that contribute to the smell of green peppers, peas and asparagus, are usually present in traditional *V. vinifera*. varieties, such as Sauvignon Blanc, Cabernet Franc, Cabernet Sauvignon, Merlot, Carmenere and Malbec. Through QTL analysis of F_1_ progeny comprising 130 genotypes for 3-isobutyl-2-methoxypyrazine (IBMP) content using solid-phase extraction (SPE) -gas chromatography-mass spectrometry (GC-MS), two genes encoding O-methyltransferases (OMTs), termed *VvOMT3* (VIT_03s0038g03090) and *VvOMT4* (VIT_03s0038g03080), on LG3 were identified to be associated with the IBMP level in grape berries (Guillaumie et al., 2013). Based on the differential expression between the high- and low-MP-producing grapevine varieties, it is proposed that *VvOMT3* is a key gene for IBMP biosynthesis in the grapevine.

However, studies on the genetic regulatory mechanism of norisoprenoids are very limited. In this study, we used an F_1_ population from the hybrid of the Muscat-type variety *V. vinifera* L. cv. Muscat Alexandria and the non-Muscat-type variety *V. vinifera* L. cv. Christmas Rose to explore the SNP loci and candidate genes significantly associated with the contents of norisoprenoid derivatives in grape berries using GWAS. The results were verified using a grapevine germplasm population and the transitent over-expression of target genes. The broader aim of this study was to gain an understanding of the genetic regulation of norisoprenoid compounds and provide a basis for the molecular breeding of grape aromas.

## 2 Materials and methods

### 2.1 F_1_ population and true hybrid identification

The F_1_ population was derived from a cross between *Vitis vinifera* L. cv. Muscat Alexandria and *V. vinifera* L. cv. Christmas Rose. The artificial hybridization was performed twice. The first hybridization was performed in 2010, and then 305 seedlings were cultivated the following year in an experimental vineyard at the Beijing Academy of Forestry and Pomology Sciences in Beijing, China (39°58′N and 116°13′E). The second hybridization was performed in 2011, and 351 seedlings were planted in an experimental vineyard in Pinggu district in Beijing, China (40°58′N and 117°52′E) in 2012. These vines were trained into a two-wire vertical trellis system with a planting space of 0.75 m × 2.5 m, and they began to bear fruits in 2015 to 2017. Similar field management was carried out for the two vineyards.

We originally selected 183 individual plants with similar growth potential from the F_1_ population and applied Kompetitive Allle-Specific Polymerase chain reaction (KASP) to distinguish true and false hybrids. Finally, 149 true fruit-bearing hybrid individuals were used for this study. The hybrid identification protocol included the following steps: First, the parents were genome re-sequenced to a depth of 30X. Second, seven different homozygous SNP loci between the two parents were selected. Finally, for each SNP locus, three pairs of primers were designed: two pairs of specific forward primers (F1 and F2) with the 3′ end carrying the mutation base of the SNP locus and the 5′ ends linked with FAM and a HEX sequencing adapter, and another pair of reverse primers (R) for general use. The length of each primer pair was about 150 bp (**Supplementary Table S1**). The three pairs of primers were mixed at an F1:F2:R:ddH_2_O ratio of 6:6:15:23. The 10 μL PCR system was composed of 1 μL DNA of an F_1_ individual, 1.4 μL mixed primers, 5 μL KASP Mix (LGC Company, UK), 0.08 μL MgCI_2_, and 2.52 μL ddH_2_O. The PCR procedure was as follows: heat treatment at 94°C for 15 min; 10 cycles of denaturation at 94°C for 20 s, annealing at 61–55°C and extension for 60 s, and 0.6°C reduction per cycle, followed by 30 cycles of denaturation at 94°C for 20 s, annealing at 55°C, and extension for 60 s. Genotyping of F_1_ individuals was conducted *via* fluorescence scanning at the end of the amplification. The heterozygous SNP was identified as the true F_1_ progeny, and the results are shown in **Supplementary Table S2**.

### 2.2 Sample collection

Young leaves of the F_1_ hybrids were sampled for re-sequencing. The grape berries were collected for norisoprenoid compound analysis when the total soluble solids (TSS) reached approximately 16°Brix in 2017, and 10°Brix and 16°Brix for the two sampling periods in 2018 (**Supplementary Figure S1**). TSS was measured using a digital handheld pocket Brix refractometer (PAL-2; ATAGO, Tokyo, Japan). When the maturity reached the sampling requirement, approximately 100 healthy berries were harvested. In the triplicate samplings, there were 126, 113, and 116 hybrid vines, respectively, that had sufficient fruits for collection. The samples were quickly frozen in liquid nitrogen and stored at –80 °C until analysis.

The leaves and fruits of a germplasm population including 97 varieties (**Supplementary Table S3**) were collected for the validation of some significant SNP loci using KASP and the determination of norisoprenoid compounds. These varieties were planted in the grape breeding nursery of Shanxi Academy of Agricultural Sciences Pomology Institute, Taigu County, Jinzhong City, Shanxi Province, China (37°42′N, 112°55′E), with a North-South row orientation and planting space of 0.5 m × 2.5 m. Three biological replicates were collected for each variety. Each replicate randomly collected 100 healthy ripe berries.

For transient over-expression analysis, the leaves of *V. quinquangularis* were collected from the Grape Research Demonstration Park of Guangxi Characteristic Crops Research Institute (Guilin, China), and wild-type tobacco (*Nicotiana benthamiana*) was cultivated in soil in a greenhouse under a cycle of 16 h light/8 h dark at 23 °C.

### 2.3 Analysis of norisoprenoids using headspace solid-phase microextraction (HS-SPME) GC-MS

The norisoprenoids in grape berry was extracted according to the previously reported method with some modification (Yuan and Qian, 2016). Approximately 70 g frozen grape berries whose seeds were removed ahead were blended with 1 g polyvinylpolypyrrolidone and 0.5 g D-gluconic acid lactone and ground into powder in liquid nitrogen. To extract norisoprenoid, 1 g berry powder was put into a 20 mL autosampler vial with 5 mL citrate buffer (0.2 M, pH 2.5, saturated with 1 g NaCl) and 10 µL internal standard (1.068 g/L 4-methyl-2-pentanol) added. Then the vials were tightly capped and equilibrated at 99 °C in a thermostatic bath for 1 h. Two technical replicates of norisoprenoid compounds detection were performed per biological replicate.

The norisoprenoid compounds were detected *via* headspace solid-phase microextraction (HS-SPME) using a CTC Combi PAL autosampler (CTC Analytics, Zwingen, Switzerland) equipped with a 2 cm DVB/CAR/PDMS 50/30 μm SPME fiber (Supelco, Bellefonte, PA., USA), and at 40°C for 30 min with stirring at 500 rpm. An Agilent 6890 gas chromatography coupled with an Agilent 5975C mass spectrometer was used to analyze the norisoprenoid compounds in the samples. The compound separation was achieved with an HP-INNOWAX capillary column (60 m 0.25 mm, 0.25 μm, J &W Scientific, Folsom, CA, USA). Norisoprenoid compounds were identified and quantified according to the previously published methods (He et al., 2021). Volatile compounds were identified by comparing their retention indices and mass spectrums using the national institute of standards and technology 11 (NIST 11) database. The standards *β*-damascenone, *β*-ionone and 6-methyl-5-hepten-2-one were used to establish standard curves in this study, whereas the other compounds without available standards were quantified using standards that had the same functional groups or similar numbers of carbon atoms (see **Table 1**). The norisoprenoids concentration was expressed in units of micrograms per kilogram.

**Table 1:** Qualitative and quantitative information of norisoprenoid compounds detected in this study.

Compounds	Molecular formula	Retention indices	Qualitative method	Quantitative compound
TCH (2,2,6-trimethylcyclohexanone)	C_9_H_16_O	1315	b	*β*-damascenone
6-methyl-5-hepten-2-one	C_8_H_14_O	1332	a	6-methyl-5-hepten-2-one
*trans*-theaspirane	C_13_H_22_O	1501	b	*β*-damascenone
vitispirane B	C_13_H_20_O	1529	b	*β*-damascenone
vitispirane A	C_13_H_20_O	1532	b	*β*-damascenone
*cis*-theaspirane	C_13_H_22_O	1540	b	*β*-damascenone
*β*-cyclocitral	C_10_H_16_O	1628	b	*β*-damascenone
riesling acetal	C_13_H_20_O	1638	b	*β*-damascenone
TDN (1,1,6-trimethyl-1,2-dihydronaphtha-lene)	C_13_H_16_	1758	b	*β*-damascenone
TPB ((*E*)-1-(2,3,6-Trimethylphenyl) buta-1,3-diene)	C_13_H_16_	1766	b	*β*-damascenone
(*Z*)-*β*-damascenone	C_13_H_18_O	1772	b	*β*-damascenone
(*E*)-*β*-damascenone	C_13_H_18_O	1834	a	*β*-damascenone
geranylacetone	C_13_H_22_O	1863	b	*β*-damascenone
*β*-ionone	C_13_H_20_O	1953	a	*β*-ionone

a: The identification of volatile compounds from full scan mode data was achieved by comparing the retention indices (RIs) and mass spectra with those of reference standards.

b: Compounds lack of standard were identified by comparing the retention indices (RIs) calculated with a C6-C24 n-alkane series under the same chromatographic conditions and mass spectrums with compounds in the NIST 11 database.

### 2.4 Whole-genome re-sequencing and SNP calling

DNA was extracted from the young leaves of the two parents and true F_1_ hybrid offspring using a Plant Total DNA Extraction Kit (Bioteke Biotechnology Company, Beijing, China). The two parents were genome re-sequenced to a depth of 30 ×, and the true F_1_ hybrid individuals were genome re-sequenced to a depth of 10 × using Illumina HiSeq platform by Annoroad Genome Technology Company (Beijing, China). Clean reads were obtained from raw reads by removing sequences with contaminated adapters, low quality, and N ratio greater than 5%. The clean reads were aligned to the *V. vinifera* Pinot noir PN 40024 reference genome (http://genomes.cribi.unipd.it/grape/) using the (Burrows-Wheeler Aligner) BWA v0.7.17 (Li and Durbin, 2009). The aligned sequences were sorted using SAMTools v1.19 (Li et al., 2009). Furthermore, PCR duplicated sequences were removed using the Picard-tools v1.119 (picard.sourceforge.net), and the sequences aligned to multiple locations were also weeded out using Perl Scripts.

All the potential SNP loci on the genome were extracted using the mutation analysis software GATK v4.0 (Genome Analysis Toolkit) (McKenna et al., 2010). Then, variants were further filtered with GATK’s variation filtration using the following filtering parameters: FS (Fisher Strand Bias) >10.0, QD (Quality by Depth) <10, DP (Coverage) <4, QUAL (Quality Scores) <30. In this study, all missing SNPs and the SNPs with the minor allele frequency (MAF) less than 0.1 have been filtered prior to the GWAS analysis using vcftools (v0.1.13) software. Finally, the high-quality SNPs were annotated according to the *V. vinifera* genome annotation file (.gff) using the ANNOVAR software (Wang et al., 2010).

### 2.5 Genome-wide association study

In this study, the norisoprenoid traits and SNP markers were used for genome-wide association study (GWAS). Manhattan plots were drawn to illustrate the significance and location distribution of SNPs for GWAS results. The significance threshold was set as -log_10_(5e-8) = 7.3 to screen out significant candidate SNPs.

In this study, we describe the measured phenotypic value *yi* of a trait for individual *i* using a regression model:


$${\text{y}}_{\text{i}}{\text{=μ+X}}_{\text{i}}{\text{β+ξ}}_{\text{i}}{\text{a+ζ}}_{\text{i}}{\text{d+ϵ}}_{\text{i}}$$

Where *μ* is the overall average value; *X_i_* refers to the subgroup to which individual *i* belongs; *β* represents the environmental factor; *a* and *d*, respectively, refer to the additive effects and dominant effects of SNPs; *ξ_i_* and *ζ_i_* refer to the indicator vectors corresponding to the additive effects and dominant effects of SNP for individual *i*; *ϵ_i_* is the residual error. The *j*-th elements of *ξ_i_* and *ζ_i_* are defined as:


$${\text{ξ}}_{\text{ij}}=\{\begin{array}{c} 1,\text{ if the genotype of SNP }j\text{ is }\mathrm{AA}\\ 0,\text{ if the genotype of SNP }j\text{ is }\mathrm{Aa}\\ -1\text{, if the genotype of SNP }j\text{ is }\mathrm{aa} \end{array}$$

$${\text{ζ}}_{\text{ij}}=\{\begin{array}{c} 1\text{, if the genotype of SNP }j\text{ is }\mathrm{Aa}\\ 0\text{, if the genotype of SNP }j\text{ is }\mathrm{AA}\text{ or }\mathrm{aa} \end{array}$$

The regression coefficients are estimated using maximum likelihood method. The hypothetical test of a marker influence on trait is formulated as follows:


$${\text{H}}_{0}\mathrm{:a}\;=0,d\text{ = }0$$

$${\text{H}}_{1}\text{: at least one of the above does not hold}$$

where *H_0_* corresponds to the simplified model and *H_1_* correspond to the full model. The test statistic of the hypothetical test is calculated as the log-likelihood ratio (LR). Level of significance is calculated by permutation test. The *p*-value thresholds of all traits were calculated through 1000 iterations using the permutation test, ultimately to determine which are significant SNP loci.

### 2.6 Screening of candidate genes

According to the location of significant SNPs on the grape reference genome (http://genomes.cribi.unipd.it/grape/), and genes within 100 kb upstream and downstream of the significant SNPs were regarded as the candidate genes. And, the focus was mainly concentrated on structural genes in the isoprenoid metabolic pathway and potential transcription factors.

### 2.7 Total RNA extraction, quantitative real-time PCR, and reverse transcription PCR

Total RNA of grape berry was extracted using the Spectrum™ Plant Total RNA Kit (Sigma-Aldrich, St. Louis, MO, USA). The quality and concentration of the obtained RNA were detected by agarose gel electrophoresis and NanoDrop 2000 spectrophotometer (Thermo Fisher Scientific, Waltham, MA, USA). First-strand cDNA was transcribed from total RNA using the HiScript ^®^ II Q RT SuperMix for qPCR + gDNA wiper Kit (Vazyme, Nanjing, China).

To detect gene expression abundance in grape berries and *V. quinquangularis* leaves, we performed quantitative real-time PCR (qRT-PCR) using ChamQ Universal SYBR qPCR Master Mix (Vazyme Nanjing, China). The PCR system is a 20 µL reaction mixture containing 2µL cDNA template, 10µL 2×ChamQ Universal SYBR qPCR Master Mix PCR, 0.8 µL primers, and 7.2 µL ddH_2_O. The PCR procedure was as follows: 95 °C for 30 s, followed by denaturation at 95 °C for 10 s, and annealing at 60 °C for 30 s, with a total of 40 cycles. The data were analyzed using the CFX Maestro™ software (Bio-Rad, USA). Three replicates were performed for each sample during qRT-PCR. The *Ubiquitin* gene was used as the reference, and the primers used for qRT-PCR are listed in **Supplementary Table S4**.

To test if the gene was expressed in *Nicotiana benthamiana* leaves, we performed the reverse-transcription PCR (RT-PCR) following published methods with some modifications (Meng et al., 2020). The 2 × Taq PCR MasterMix (KT201) (Tiangen Biotech, Beijing, China) was used for PCR, wherein a 10 µL reaction mixture contained 1 µL of the cDNA template, 1 µL primers, and 3 µL ddH_2_O. The *NbL23* gene was used as an internal control. The PCR procedure was as follows: 94 °C for 5 min, 94 °C for 30 s, 56 °C for 30 s, and 72 °C for 60 s, followed by a final extension step for 5 min at 72°C, with a total of 29 cycles. The products were visualized through agarose gel electrophoresis. Primers used for RT-PCR are listed in **Supplementary Table S4**.

### 2.8 *VvGGPPS* function validation by transient overexpression in *V. quinquangularis* leaves

The transient transformation of *VvGGPPS* (VIT_205s0020g01240) was carried out in the leaves of *V. quinquangularis* using published methods with some modifications (Xu et al., 2010). First, the coding region of the gene was amplified from the cDNA of *V. vinifera* ‘Petit Manseng’ grapes with one pair of primers (**Supplementary Table S4**), and then sequenced. Next, the full-length coding fragment was cloned into the pCAMBIA1301 vector, and this construct was under the control of the CaMV 35S promoter. The expression construct pCAMBIA 1301-*VvGGPPS* was transferred into the *Agrobacterium tumefaciens* strain EHA105, and the empty pCAMBIA 1301 vector was used as the control. Subsequently, the activated *A. tumefaciens* EHA105 strain carrying the plasmid pCAMBIA 1301-*VvGGPPS* was inoculated into 50 mL LB liquid medium (20 mg/L rifampicin and 50 mg/L kanamycin) to attain the initial optical density at 600 nm (OD_600_) of approximately 0.2, and then cultured on a shaker of 220 rpm at 28 °C until the OD600 reached 1.0–1.2. After the centrifugation at 4 °C and 4,000 × g for 5 min, the strains were resuspended with induction buffer consisting of 2.132 g/L MES (2-(N-morpholino) ethanesulfonic acid), 2.033 g/L MgCl_2_·6H_2_O, 5 g/L sucrose, and 0.039 g/L acetosyringone (pH 5.9–6.0) to obtain the final OD600 value of 0.4. The re-suspensended solution was maintained at 25 °C for 3 h to adapt the strains to the new buffer environment. Finally, the leaves backside up were submerged into the adjusted solution and subjected to vacuum infiltration (–0.8 MPa) for 20 min. Both the control group and the overexpression group were set three replicates with two leaves per replicate. After 3 days of incubation at 25 °C in the dark, the leaves were used for gene expression analysis and norisoprenoids detection. The relative gene expression levels were analyzed *via* qRT-PCR and the norisoprenoid compounds were measured *via* HS- SPME-GC-MS as mentioned above.

### 2.9 *VvGGPPS* function validation by transient overexpression in *N. benthamiana* leaves

In contrast to the vacuum infiltration method described above, the transient overexpression in *N. benthamiana* leaves was performed using the injection method (Meng et al., 2020). The activated *A. tumefaciens* EHA105 strain carrying the plasmid pCAMBIA 1301-*VvGGPPS* was inoculated into 50 mL LB liquid medium (20 mg/L rifampicin and 50 mg/L kanamycin) to attain the initial OD_600_ of approximately 0.2, and then cultured on a shaker of 220 rpm at 28 °C until the OD_600_ reached 1.0–1.2. After the centrifugation at 4 °C and 4,000 × g for 5 min, the strains were resuspended with induction buffer consisting of 11.92 g/L MES, 20.33 g/L MgCl_2_·6H_2_O, and 19.62 g/L acetosyringone (pH 5.7) to obtain the final OD_600_ value of 0.6–0.8. The re-suspensended solution was maintained at 25 °C for 3 h. The *A. tumefaciens* solution containing the pCAMBIA 1301 empty vector was used as the the control. The strain solution was injected with a syringe without a needle into the leaves of *N. benthamiana* of about 5 weeks of age. Subsequently, these plants were cultured in the dark for 12 h and then transferred to the greenhouse for normal culture for 3 days. The leaves were collected for gene expression analysis and determination of aroma compounds. Both the control and transient overexpression groups were injected with at least two plants, totaling four leaves per replicate, and the experiments in each group had three replicates.

### 2.10 Data analysis

The SPSS version 22.0 (SPSS Corp., Armonk, NY, USA). was used for all significance analysis at *p* < 0.05 (Duncan’s multiple range test or *T*-test). Figures were drawn using the GraphPad Prism 8.0.2 (GraphPad Software, USA). Pearson’s correlation coefficient calculations and heatmaps were generated using the “psych” and “corrplot” packages in R software (v 3.6.2).

## 3 Results

### 3.1 Variation of norisoprenoids concentration in berries of the F_1_ hybrid population

There were 14 kinds of norisoprenoid compounds qualitatively identified in the detected grape berries of the hybrid generation (**Table 1**), and they were individually quantified according to the standard curves. To facilitate association analysis, the concentrations of isomers *trans*- and *cis*- theaspirane, vitispirane A and B, as well as (*Z*)- and (*E*)-*β*-damascenone, respectively, were summed up as theaspirane, vitispirane and *β*-damascenone traits. In addition, the concentrations of norisoprenoid derivatives with 13 carbons and all the detected compounds, respectively, were also summed up as C13-norisoprenoid and total norisoprenoid traits. In the present study, C13-norisoprenoid derivatives included theaspirane, vitispirane, riesling acetal, TDN, TPB, *β*-damascenone and geranylacetone. As a result, a total of 13 norisoprenoid traits were considered in subsequent analysis (**Table 2**).

**Table 2:** Statistical analysis of norisoprenoids concentrations in the F_1_ hybrid population.

Norisoprenoids	2017 16°Brix				2018 10°Brix				2018 16°Brix			
	Mean (μg/kg)	Minimum (μg/kg)	Maximum(μg/kg)	CV	Mean (μg/kg)	Minimum (μg/kg)	Maximum (μg/kg)	CV	Mean (μg/kg)	Minimum (μg/kg)	Maximum (μg/kg)	CV
TCH (2,2,6-trimethylcyclohexanone)	0.36	0.16	0.75	20%	2.73	1.10	5.34	29%	2.74	1.56	4.57	22%
6-methy-5-hepten-2-one	9.84	0.97	50.10	91%	7.51	3.98	20.92	40%	6.58	3.98	17.20	39%
theaspirane	0.60	0.58	0.67	3%	0.43	0.04	2.31	87%	0.23	0.04	0.91	74%
vitispirane	1.70	0.42	6.84	67%	15.10	7.26	39.64	45%	20.08	9.19	63.86	48%
*β-*cyclocitral	0.31	0.15	2.07	69%	1.88	0.62	8.59	64%	2.38	0.75	9.32	77%
riesling acetal	0.28	0.15	1.49	60%	1.84	1.24	3.48	22%	2.68	1.37	8.55	48%
TDN(1,1,6-trimethyl-1,2-dihydronaphtha-lene)	2.18	0.37	10.52	81%	10.99	5.51	44.83	58%	19.75	6.93	70.13	69%
TPB((E)-1-(2,3,6-Trimethylphenyl) buta-1,3-diene)	0.21	0.15	0.40	23%	1.21	0.10	4.28	61%	1.78	0.41	5.36	55%
*β*-damascenone	41.47	13.77	96.84	37%	11.37	6.06	23.48	29%	13.23	6.13	27.79	32%
geranylacetone	0.25	0.00	1.11	83%	1.75	0.71	3.99	42%	1.46	0.68	3.42	37%
*β-*ionone	0.16	0.14	0.20	70%	1.07	0.40	2.60	38%	0.59	0.06	1.14	32%
C13-norisoprenoids	46.84	16.11	105.68	35%	43.76	23.62	116.26	35%	59.79	28.63	165.94	42%
Total norisoprenoids	57.35	18.18	114.01	30%	55.87	29.53	126.58	28%	71.49	37.64	182.06	36%

CV refers to the coefficient of variation of compound concentration among F_1_ hybrid individuals.

The concentrations of total norisoprenoids ranged from 18.18 to 114.01 μg/kg, 29.53 to 126.58 μg/kg and 37.64 to 182.06 μg/kg at the three sampling periods, with mean values of 57.35 μg/kg, 55.87 μg/kg, and 71.49 μg/kg, respectively (**Table 2**). The compound *β*-damascenone had the highest concentration, accounting for approximately 63% of the total norisoprenoids in 2017. Besides, TDN and vitispirane also contributed a substantial proportion to the total norisoprenoids concentration detected in 2018. Overall, the concentration of norisoprenoids in the grape berries in 2018 was higher than that in 2017, suggesting that vintage had a large effect on the accumulation of norisoprenoid compounds. Therefore, it is necessary to use data from multiple sampling points to study complex quantitative traits that are greatly affected by environmental factors. Certainly, we also found that the concentrations of some compounds varied slightly between the two years, such as *β*-ionone and theaspirane, but both of them showed a relatively low levels in the hybrid generation. From the data of the two sampling periods in 2018, it was found that the concentrations of *β*-ionone, MHO, geranylacetone and theaspirane decreased with berry ripening, whereas those of other norisoprenoid compounds increased (**Table 2**).

**Figure 1:**
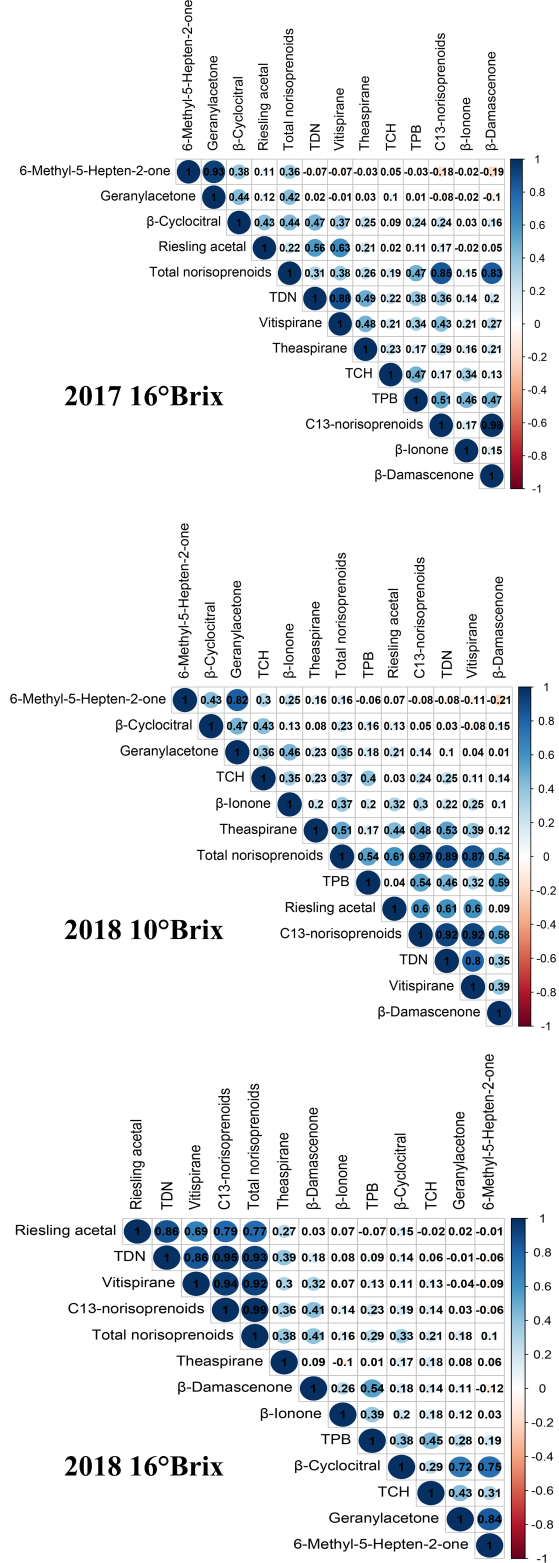
Correlations among the concentrations of various norisoprenoid compounds in the F_1_ population at three sampling periods. The numbers in the graph are correlation coefficients, and the significant correlation (*p* < 0.01) are highlighted with colored circles (blue and red). The color intensity of the circle also indicates the strength of the relationship.

With the exception of the variation in theaspirane being less than 10% in 2017, the variations of the other norisoprenoid traits in the F_1_ population were between 10% and 100%, which are considered moderate variations (**Table 2**). Among them, the compounds with large variations in 2017 were MHO, geranylacetone and TDN, reaching 91%, 83%, and 81%, respectively, whereas the largest two variations in 2018 occurred in theaspirane (87%) and *β*-cyclocitral (77%). Comparing the variation amplitude of individual norisoprenoid traits between the two experimental years, it can be concluded that various norisoprenoid traits all had a wide and continuous segregation in the hybrid generation of grapevines. Furthermore, the association between different norisoprenoid traits was assessed using Pearson correlation analysis. A positive correlation was found among the traits of 6-methyl-5-hepten-2-one, geranylacetone and *β*-cyclocitral with the coefficients of 0.38–0.93 (**Figure 1**). Moreover, there was also a positive correlation among the concentrations of TDN, vitispirane and riesling acetal with the coefficients of 0.56–0.88 (**Figure 1**). These results indicated that there may be a similar regulatory mode involved in manipulating the biosynthesis of these traits.

### 3.2 Screening of trait-associated SNP loci using GWAS

In the present study, 13 norisoprenoid content traits and 568,953 filtered SNPs were used for GWAS analysis.

**Figure 2:**
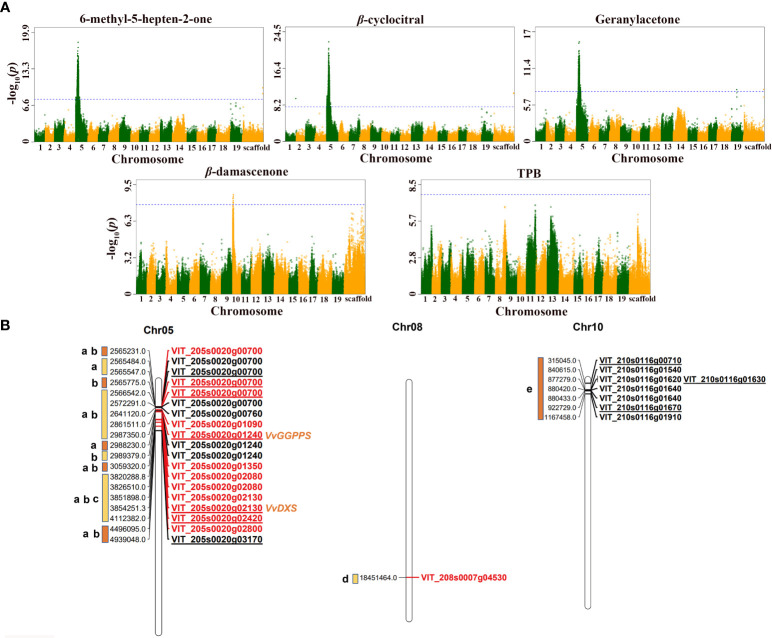
Genome-wide association analysis of five norisoprenoid traits in grape berry. **(A)** Manhattan plots of five norisoprenoid traits. The x-axis represents the position along a chromosome and the y-axis refers to the -log_10_ (*P*-value). Each point is the -log_10_ (*P*-value) of a SNP locus. The blue horizontal dashed line depicts the significance threshold, -log_10_ (5e-8) =7.3. **(B)** Chromosomal location of main candidate genes. Different lowercase letters represent different traits: a, b, c, d, and e represent 6-methyl-5-hepten-2-one, *β*-cyclocitral, geranylacetone, TPB, and *β*-damascenone, respectively. The gene ID in red indicates that the gene has been verified later, and the underlined gene ID indicates that the locus was located in the coding region of the gene.

The results of GWAS revealed that, except for *β*-ionone, theaspirane and TCH, the remaining 10 traits were all associated with significant SNP loci. Overall, the significant SNP loci associated with MHO, *β*-cyclocitral and geranylacetone were all located on chromosome 5 (**Figures 2A, B**), and the significant SNP loci related to *β*-damascenone were located on chromosome 10 (**Figures 2A, B**). In addition, most of significant SNP loci associated with TDN, vitispirane and riesling acetal were distributed on chromosome 11, and only a few were scattered on chromosome 18, 19, and 20 (**Supplementary Figure S2**). There were only two SNPs on chromosome 8 showing significant association with TPB (**Figures 2A, B**). The significant SNP loci associated with C13-norisoprenoids and total norisoprenoids were concentrated on chromosome 11 (**Supplementary Figure S2**). It was particularly notabled that many SNP loci associated with MHO, geranylacetone, and *β*-cyclocitral were identical. Similarly, many significant loci related to TDN, vitispirane, and riesling acetal were also the same. This phenomenon echoes the strong or moderate correlations found among the phenotypic traits described above (see **Figure 1**).

Specifically, there were 1383, 2244, and 2497 significant SNP loci individually associated with geranylacetone, MHO, and *β*-cyclocitral, respectively, 138, 238, and 284 of which were located in the coding sequence (CDS) region of genes. Furthermore, most of the loci and candidate genes related to these three traits were identical (**Supplementary Table S5**). The identical candidate genes included *VvDXS1* (VIT_205s0020g02130) and *VvGGPPS* (VIT_205s0020g01240) at the upstream of the norisoprenoid biosynthetic pathway, lipoxygenase, *VvLOX* (VIT_205s0020g03170), pyruvate dehydrogenase kinase (VIT_205s0020g00760), carbohydrate kinase (VIT_205s0020g02800), and the others (**Table 3**). The SNP loci at the chr5:3854251, chr5:2987350, and chr5:4939048 were located in the CDS regions of *VvDXS1*, *VvGGPPS*, and *VvLOX*, respectively. In addition, it was found that there were significant SNP loci inside the genes encoding transcription factors, the loci at chr5:2565547 and chr5:2565775 were in the CDS regions of squamosa promoter-binding like protein 1 (VIT_205s0020g00700) and chr5:4112382 was in the CDS regions of BSD domain-containing protein (VIT_205s0020g02420).

**Table 3:** Information of candidate significant SNP loci within genes and their gene annotation.

Norisoprenoids	SNP loci	*p*-value	Gene annotation	Gene ID	Location of SNPs in genes
6-methyl-5-hepten-2-one	chr5:2565484	1.00E-09	squamosa promoter-binding like protein 1 (SPL)	VIT_205s0020g00700	3’UTR
6-methyl-5-hepten-2-one	chr5:2565547	1.60E-08	squamosa promoter-binding like protein 1 (SPL)	VIT_205s0020g00700	CDS
6-methyl-5-hepten-2-one	chr5:2988230	7.13E-11	GGPPS (geranylgeranyl diphosphate synthase)	VIT_205s0020g01240	5’UTR
*β-*cyclocitral	chr5:2565775	9.67E-09	squamosa promoter-binding like protein 1 (SPL)	VIT_205s0020g00700	CDS
*β-*cyclocitral	chr5:2989379	9.73E-10	GGPPS (geranylgeranyl diphosphate synthase)	VIT_205s0020g01240	intron
6-methyl-5-hepten-2-one, *β*-cyclocitral	chr5:2565231	2.45E-10	squamosa promoter-binding like protein 1 (SPL)	VIT_205s0020g00700	3’UTR
6-methyl-5-hepten-2-one, *β*-cyclocitral	chr5:2566542	1.51-E11	squamosa promoter-binding like protein 1 (SPL)	VIT_205s0020g00700	CDS
6-methyl-5-hepten-2-one, *β*-cyclocitral	chr5:2572291	1.39E-11	squamosa promoter-binding like protein 1 (SPL)	VIT_205s0020g00700	5’UTR
6-methyl-5-hepten-2-one, *β*-cyclocitral	chr5:2641120	1.73E-10	pyruvate dehydrogenase kinase (PDK)	VIT_205s0020g00760	intron
6-methyl-5-hepten-2-one, *β*-cyclocitral	chr5:2861511	4.49E-09	bZIP protein HY5	VIT_205s0020g01090	3’UTR
6-methyl-5-hepten-2-one, *β*-cyclocitral	chr5:2987350	5.10E-12	GGPPS (geranylgeranyl diphosphate synthase)	VIT_205s0020g01240	CDS
6-methyl-5-hepten-2-one, *β*-cyclocitral	chr5:3059320	1.12E-10	Nuclear transcription factor Y subunit B-5	VIT_205s0020g01350	3’UTR
6-methyl-5-hepten-2-one, *β*-cyclocitral	chr5:4496095	1.15E-11	carbohydrate kinase	VIT_205s0020g02800	3’UTR
6-methyl-5-hepten-2-one, *β*-cyclocitral	chr5:4939048	3.95E-10	linoleate 9S-lipoxygenase 5 (LOX)	VIT_205s0020g03170	CDS
6-methyl-5-hepten-2-one, geranylacetone, *β*-cyclocitral	chr5:3820289	3.12E-12	zinc finger proteins (ZFP)	VIT_205s0020g02080	3’UTR
6-methyl-5-hepten-2-one, geranylacetone, *β*-cyclocitral	chr5:3826510	2.85E-13	zinc finger proteins (ZFP)	VIT_205s0020g02080	intron
6-methyl-5-hepten-2-one, geranylacetone, *β*-cyclocitral	chr5:3851898	4.42E-16	DXS1(1-deoxy-D-xylulose-5-phosphate synthase1)	VIT_205s0020g02130	CDS
6-methyl-5-hepten-2-one, geranylacetone, *β*-cyclocitral	chr5:3854251	4.18E-18	DXS1(1-deoxy-D-xylulose-5-phosphate synthase1)	VIT_205s0020g02130	intron
6-methyl-5-hepten-2-one, geranylacetone, *β*-cyclocitral	chr5:4112382	2.70E-13	BSD domain-containing protein	VIT_205s0020g02420	CDS
*β-*damascenone	chr10:315045	4.65E-09	tryptophan synthase (TS)	VIT_210s0116g00710	CDS
*β-*damascenone	chr10:840615	3.98E-09	aminomethyltransferase	VIT_210s0116g01540	5’UTR
*β-*damascenone	chr10:877279	2.77E-09	rhamnogalacturonate lyase (RGL)	VIT_210s0116g01620	3’UTR
*β*-damascenone	chr10:877279	2.77E-09	endonuclease/exonuclease/phosphatase family (EEP)	VIT_210s0116g01630	CDS
*β-*damascenone	chr10:880420	1.03E-08	glucan endo-1,3-beta-glucosidase	VIT_210s0116g01640	3’UTR
*β-*damascenone	chr10:880433	1.03E-08	glucan endo-1,3-beta-glucosidase	VIT_210s0116g01640	3’UTR
*β-*damascenone	chr10:922729	8.41E-09	prephenate dehydratase (PDT)	VIT_210s0116g01670	CDS
*β-*damascenone	chr10:1167458	2.03E-09	ABC transporter	VIT_210s0116g01910	intron
TPB	chr8:18451465	1.42E-08	carotene *ϵ*-monooxygenase (LUT1)	VIT_208s0007g04530	3’UTR

UTR, untranslated regions; CDS, coding sequence.

The colored and highlighted values are p-values. P-values indicates the significance level of correlation between SNP loci and norisoprenoid traits.

There were ten significant SNP loci associated with *β*-damascenone. Of them, three loci at chr10:315045, chr10:877279, and chr10:922729, were found in the CDS regions of tryptophan synthase (VIT_210s0116g00710), endonuclease/exonuclease/phosphatase family (VIT_210s0116g01630), and prephenate dehydratase (VIT_210s0116g01670), respectively (**Table 3**). In particular, the locus at chr10:922729 was the lead SNP showing the significant association with *β*-damascenone with the *p*-value of 9.67 × 10^-8^. Of the two significant SNP loci associated with TPB, one at chr8:18451465 was in the 3′- untranslated region (UTR) of the carotene *ϵ*-monooxygenase (VIT_208s0007g04530) (**Table 3**), and the other at chr8:18552647 was in the intron region of the SHOOT GRAVITROPISM 6 (SGR6) isoform X1(VIT_208s0007g04630) (**Supplementary Table S6**). It is speculated that carotene *ϵ*-monooxygenase (VIT_208s0007g04530) may be involved in the synthesis of TPB (**Table 3**).

The significant SNP loci within genes associated with other norisoprenoid traits are listed in **Supplementary Table S6**. Of the 634 significant SNP loci associated with vitispirane, there were 232 loci within the genes, of which 20 were in the CDS region. These SNP loci were mapped to 43 genes. The 98 significant SNP loci related to riesling acetal were mapped to 13 genes in total. Among them, four of the 33 SNP loci within the genes existed in the CDS region. As for TDN, there were 130 of 368 associated SNP loci within genes, of which nine were in the CDS region. A total of 40 and 27 SNP loci showed significant association with C13-norisoprenoid and total norisoprenoid traits, respectively, corresponding to five and three genes. Of them, there were eight and four SNP loci, respectively, inside the genes. With regard to the candidate genes listed in **Supplementary Table S6**, we did not pay more attention to them because so far there is no evidence that they are involved in the biosynthesis and regulation of norisoprenoid compounds.

Considering that the 19 significant SNP loci associated with MHO, *β*-cyclocitral and geranylacetone were all concentrated in the upstream region of chromosome 5 (see **Figure 2B**), we performed the haplotype analysis of these SNPs using haploview4.2 software to assess the linkage disequilibrium (LD) between them. The result showed that there were two large LD-blocks: one with 8 haplotypes (Hap1-8), and the other with 7 haplotypes (Hap 9-15) (**Supplementary Figure S6**). Hap1 and Hap 9 were the two major haplotypes, accounting for 62.3% and 66.9%, respectively. Furthermore, the two major haplotypes were found to be strongly associated with low phenotype concentration. The Hap 1 was composed of the alleles of the 12 markers (C-T-T-T-G-C-G-G-C-C-A-C), and the markers 9-11 (C-C-A) corresponded to three SNPs within the *VvGGPPS* gene at chr5:2987350, chr5:2988230 and chr5:2989379. The Hap 9 was composed of the alleles of the 5 markers (C-C-A-C-A), and the markers 15-16 (A-C) corresponded to the two SNPs within the *VvDXS* gene at chr5:3851898 and chr5:3854251.

### 3.3 Genotype analysis of candidate SNP loci in the F_1_ population

**Figure 3:**
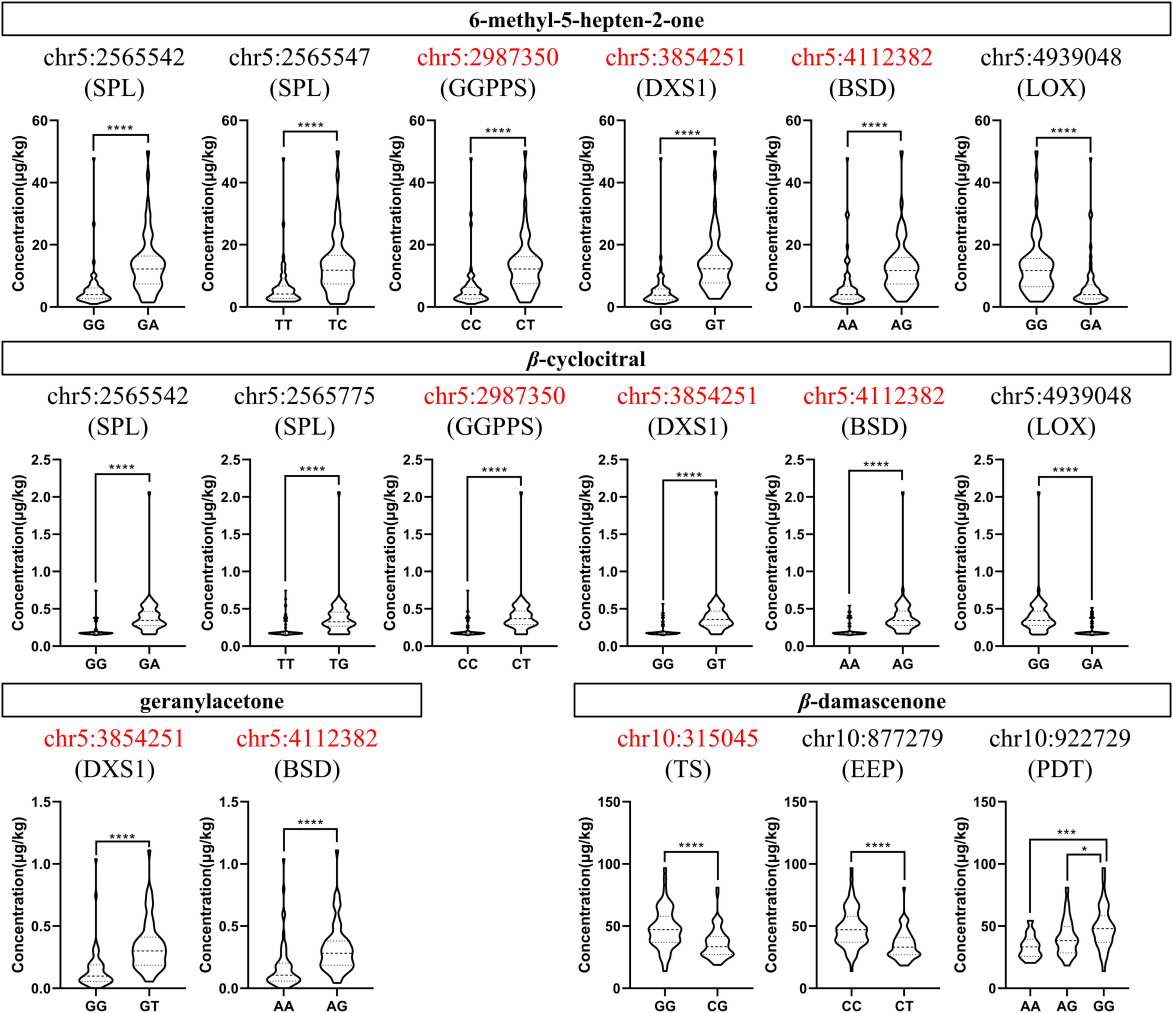
Concentration distribution violin plots of different genotypes at candidate SNP loci located in the coding sequence region of the genes in the F_1_ population when the TSS was 16°Brix in 2017. The capital letters below the SNP loci are the symbols of the genes which these loci are located in. The SNP loci highlighted in red represent non-synonymous mutations. SPL, squamosa promoter-binding like protein 1; GGPPS, geranyl-geranyl diphosphate synthase; DXS1, 1-deoxy-D-xylulose-5-phosphate synthase1; BSD, BSD domain-containing protein; LOX, linoleate 9S-lipoxygenase 5; TS, tryptophan synthase; EEP, endonuclease/exonuclease/phosphatase family; PDT, prephenate dehydratase. The asterisks *** and **** indicate significant levels at *p*<0.001 and *p*<0.0001, respectively. Concentration distribution violin plots of different genotypes at candidate SNP loci located in the coding sequence region of the genes in the F_1_ population when the TSS was 10°Brix and in 16°Brix in 2018 were shown in **Supplementary Figures S3**, **S4**, respectively. The asterisks * indicates significant levels at *p*<0.05.

We focused on the genotype analysis of 27 candidate SNP loci listed in **Table 3**. It was found that most of the SNP loci in the F_1_ population had only two genotypes, and there were significant differences in the compound concentration traits between the genotypes, as expected. Among the candidate loci, 10 SNPs were found in the CDS region of the genes. In particular, four loci of them were non-synonymous mutations. We further analyzed the genotypes of these 10 loci and their corresponding traits (**Figure 3**; **Supplementary Figures S3**, **S4**). It was found that the mutation from C to T at chr5:2987350 would result in the substitution of glycine (G) with arginine (R) at position 261 of VvGGPPS (VIT_205s0020g01240), and this mutation could increase the levels of MHO and *β*-cyclocitral in grape berries. The substitution of lysine (K) with asparagine (N) at position 284 of VvDXS1 (VIT_205s0020g02130), due to the mutation of G to T at chr5:3854251, also elevated the levels of MHO, *β*-cyclocitral and geranylacetone.

In addition to the two genes of the isoprenoid metabolism, the other two SNP loci with non-synonymous mutations were found at chr5:4112382 and chr10:315045, corresponding to A/G and C/G mutations, respectively, which replaced serine (S) into asparagine (N) at position 33 of the BSD domain-containing protein (VIT_205s0020g02420) and glycine (G) into alanine (A) at position 28 of the tryptophan synthase (VIT_210s0116g00710). Overall, the grape berries with genotype A/G at chr5:4112382 had higher concentrations of MHO, *β*-cyclocitral and geranylacetone than those with genotype A/A, and the grape berries with genotype G/G at chr10:315045 displayed higher *β*-damascenone concentration than those with genotype C/G. Except these four SNP loci, the remaining six ones all showed synonymous mutations, but different genotypes still presented significant differences in the norisoprenoid traits (**Figure 3**).

### 3.4 Validation of candidate SNP loci in the germplasm population using KASP

**Figure 4:**
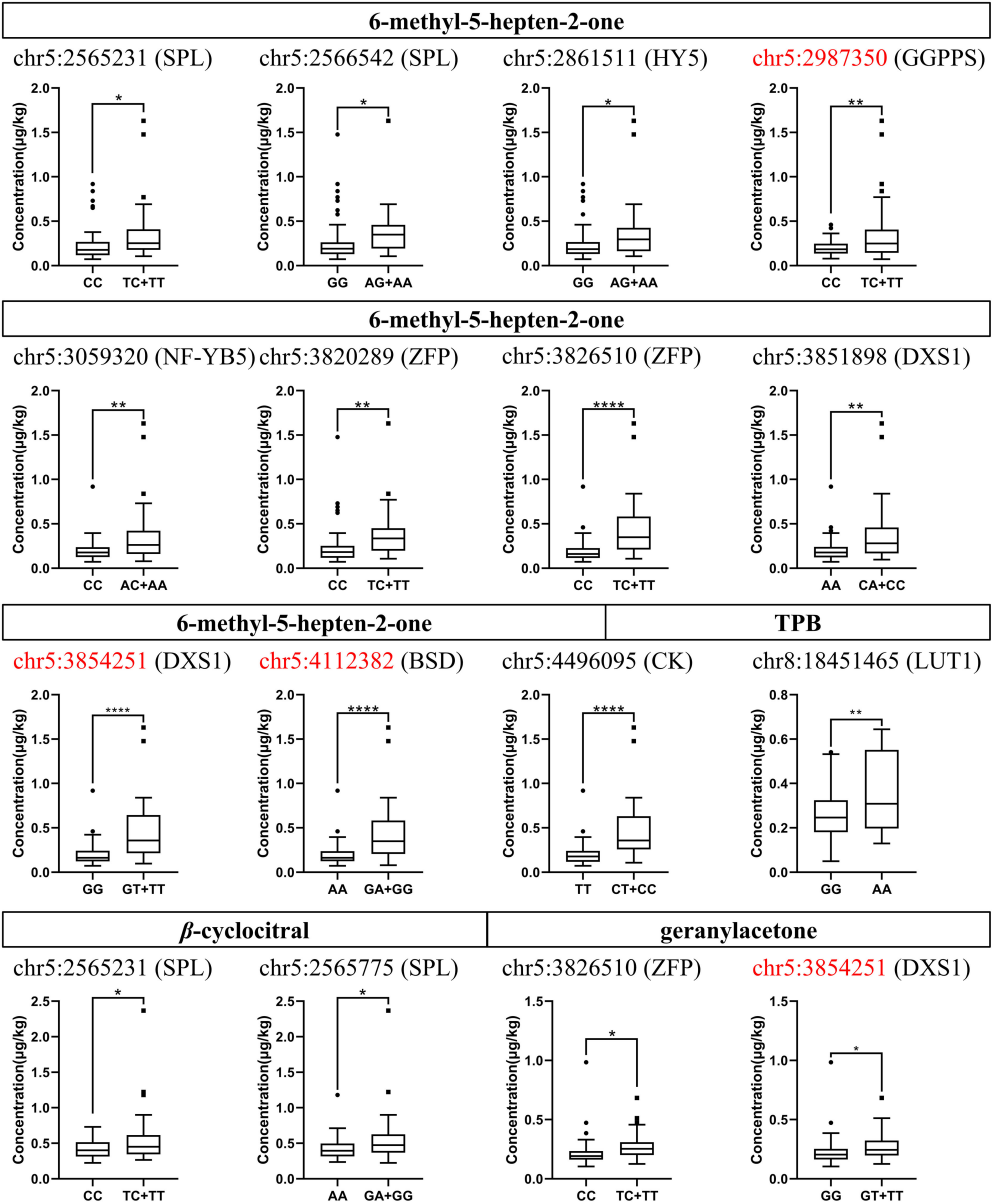
Concentration distribution box plots of different genotypes at candidate SNP loci in the grapevine germplasm population. The concentration ranges in the vertical coordinates are the compound concentration data obtained from 97 individuals. The capital letters to the right of the SNP loci are the symbols of the genes which these loci are located in. The SNP loci highlighted in red represent non-synonymous mutations. SPL, squamosa promoter-binding like protein 1; HY5, bZIP protein HY5; GGPPS, geranyl-geranyl diphosphate synthase; NF-YB5, Nuclear transcription factor Y subunit B-5; ZFP, zinc finger proteins; DXS1, 1-deoxy-D-xylulose-5-phosphate synthase; BSD, BSD domain-containing protein; CK, carbohydrate kinase; LUT1, carotene *ϵ*-monooxygenase. The asterisks *, **, and ****, indicate significant levels at *p*<0.05, *p*<0.01 and *p*<0.0001, respectively.

To validate the above GWAS results, we further examined 27 candidate SNP loci (**Table 3**) in a grapevine germplasm population comprising 97 varieties using KASP. The KASP primers used are listed in **Supplementary Table S7**. Combined the results of the genotypes and the corresponding norisoprenoid traits, 13 SNP loci were confirmed. Of them, 11 SNP loci were related to 6-methyl-5-hepten-2-one, two were related to *β*-cyclocitral, and two were related to geranylacetone, and one was related to TPB (**Figure 4**). In the grapevine germplasm population, it was alike found that the germplasm individuals with the T/C or T/T genotype at chr5:2565231 had higher average levels of 6-methyl-5-hepten-2-one than those with the C/C genotype, while the individuals with G/A or G/G at chr5:2565775 had a higher average level of *β*-cyclocitral compared to these with the A/A. The remaining 11 SNP loci were also consistent with the association results obtained using GWAS, which will not be repeated here. It should be emphasized that three of the four non-synonymous mutations SNP loci mentioned above were validated as well, which were the loci at chr5:2987350, chr5:3854251, and chr5:4112382.

In the germplasm population, the proportions of the three genotypes at chr5:2987350 in the CDS region of *VvGGPPS* were 59% T/C, 34% C/C, and 7% T/T. The individuals with the T/C or T/T genotypes displayed a wide concentration distribution of MHO, but consistently showed a higher average level than those of individuals with the C/C genotype. The three genotypes of chr5:3854251 in the CDS region of *VvDXS1* presented the genotype distribution of 63% G/G, 30% G/T, and 7% T/T, respectively, and the individuals with the G/T or T/T genotype had higher average levels of MHO and geranylacetone compared with those of individuals with G/G. As for the SNP locus at chr5:4112382 in the CDS region of BSD domain-containing like protein, the three genotypes individually accounted for 55% A/A, 31% G/A and 14% G/G, and the average concentration of MHO in the individuals with the G/A or G/G genotype was higher than that of individuals with the A/A genotypes (**Supplementary Table S8**; **Figure 4**).

### 3.5 Expression of *VvDXS1* and *VvGGPPS* in F_1_ hybrid individuals

**Figure 5:**
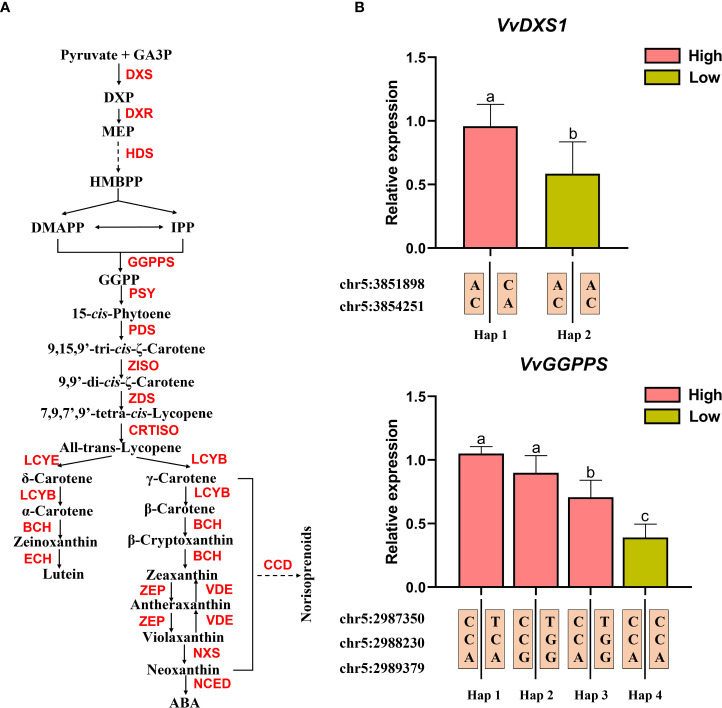
Gene expression levels of *VvGGPPS* and *VvDXS1* in individuals with extreme pools. **(A)** Pathway of isoprenoid metabolism. GA3P, D-glyceraldehyde-3-phosphate; DXP, 1-deoxy-D-xylulose-5-phosphate; MEP, methyl-erythritol-phosphate; HMBPP, hydroxymethylbutenyl diphosphate; DMAPP, dimethylallyl diphosphate; IPP, iso- pentenyl diphosphate; GGPP geranylgeranyl diphosphate; ABA, Abscisic acid; DXS, 1-deoxy-D-xylulose-5-phosphate synthase 1; DXR, 1-deoxy-D-xylulose-5-phosphate reductoisomerase; HDS, 4-hydroxy-3-methylbut-2-enyl diphosphate synthase; GGPPS, geranyl-geranyl diphosphate synthase; PSY, phytoene synthase; PDS, phytoene desaturase; ZISO, *ζ*-carotene isomerase; ZDS, *ζ*-carotene desaturase; CRTISO, carotene isomerase; LCYB, lycopene *β*-cyclase; LCYE, lycopene *ϵ*-cyclase; BCH, *β*-carotene hydroxylase; ECH, *ϵ*-carotene hydroxylase; ZEP, zeaxanthin epoxidase; VDE, violaxanthin de-epoxidase; NXS, neoxanthin synthase; NCED, 9-*cis*-epoxycarotenoid dioxygenase; CCD, carotenoid cleavage dioxygenase. **(B)** Gene expression levels of *VvGGPPS* and *VvDXS1* in high- and low- concentration individuals and the haplotypes of the high- and low- concentration individuals at these SNP loci.

Both *VvDXS1* (VIT_205s0020g02130) and *VvGGPPS* (VIT_205s0020g01240) are involved in the isoprenoid metabolism and act at the upstream of norisoprenoid biosynthesis (**Figure 5A**). A total of five candidate significant SNP loci were located within these two genes (see **Table 3**). The above results demonstrated that the candidate significant SNP loci in the CDS regions of the two genes were non-synonymous mutations and significantly associated with MHO, *β*-cyclocitral, and geranylacetone levels. To this end, we selected six F_1_ individuals related to the above three traits for gene expression analysis, three with low concentration of indicated compound and three with high concentrations, and showing two different genotypes at the SNP loci in their CDS regions, to understand whether the mutation of genotype would affect the expression of the gene. The information about the selected F_1_ individuals and the three norisoprenoid compound concentrations is shown in the **Supplementary Table S9**. The results showed that both *VvDXS1* and *VvGGPPS* had higher expression levels in the individuals with high concentrations than in the individuals with low-concentrations. It was suggested that the mutations at chr5:3851898, chr5:3854251 and chr5:2987350 might affect the expression of *VvDXS1* and *VvGGPPS*. As mentioned above, the two high frequency haplotypes (Hap1 and Hap 9) were associated with the low levels of MHO, *β-*cyclocitral, and geranylacetone (**Supplementary Figure 6**). In this context, we also found that two other SNP loci at chr5:2988230 and chr5:2989379 in the 5′-UTR and intron of the *VvGGPPS* gene, respectively, and their genotypes did not have an exact association with either the compound concentration traits or the gene expression levels (**Figure 5B**).

### 3.6 Functional validation of *VvGGPPS* in transient expression system

Based on the above results, we mainly investigated the function of *VvGGPPS* (VIT_205s0020g01240) in controlling the production of 6-methyl-5-hepten-2-one and *β*-cyclocitral using homologous and heterologous transient transformation systems.

**Figure 6:**
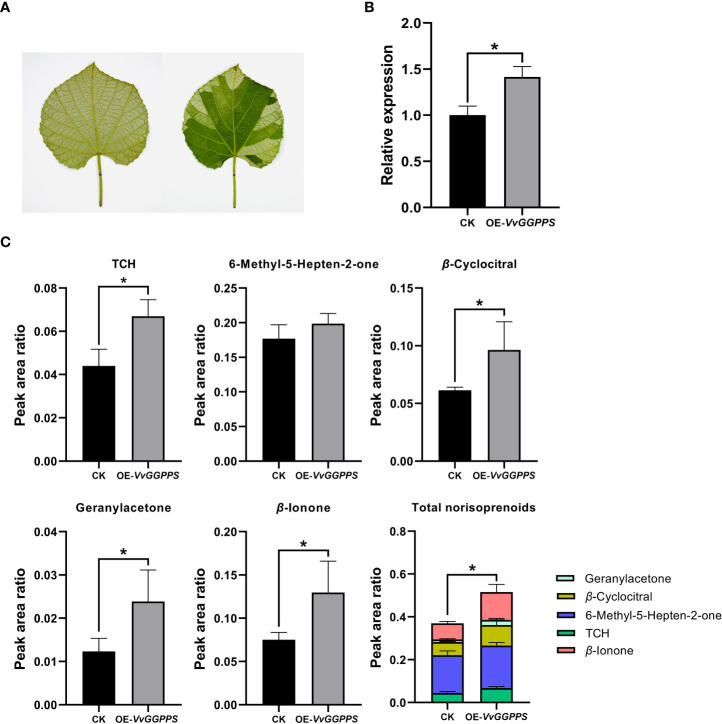
Functional analysis of *VvGGPPS* in the leaves of *Vitis quinquangularis*. **(A)** Pictures displaying uninfected (left) and infected (right) leaves of *V. quinquangularis.* **(B)** qRT–PCR analysis of *VvGGPPS* expression in the leaves of *V. quinquangularis.*; *Ubquintin* was used as the internal control. **(C)** Concentrations of norisoprenoid compounds detected in leaves of *V. quinquangularis*. CK represents the control groups from uninfected leaves; OE-*VvGGPPS* represents the overexpressed *VvGGPPS* groups from infected leaves. The asterisks * indicates significant levels at *p*<0.05.

**Figure 7:**
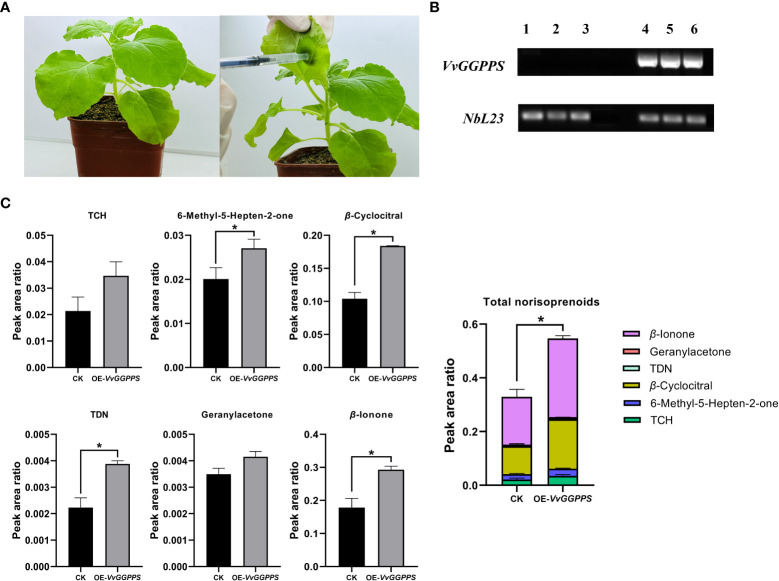
Functional analysis of *VvGGPPS* in the leaves of tobacco (*Nicotiana benthamiana*). **(A)** Pictures displaying normal wild-type (left) and injected (right) leaves of *N. benthamiana.* **(B)** RT–PCR analysis of *VvGGPPS* expression. The original figure is shown in **Supplementary Figure S5**. *NbL23* was used as the internal reference gene. Lanes 1 to 3 on the electrophoretic gel represent control groups; Lanes 4 to 6 on the electrophoretic gel represent overexpressed *VvGGPPS* groups. **(C)** Concentrations of norisoprenoid compounds detected in the leaves of tobacco. CK represents the control groups from wild-type leaves; OE-*VvGGPPS* represents the overexpressed *VvGGPPS* groups from injected leaves. The asterisks * indicates significant levels at *p*<0.05.

Using the *Agrobacterium*-mediated transient expression system in the leaves of *V. quinquangularis* (**Figure 6A**), *VvGGPPS* was successfully over-expressed by approximately1.5-fold (**Figure 6B**). The qualitative and quantitative analyses of the norisoprenoids in the leaves indicated that five norisoprenoid derivatives were identified and four of them were significantly increased, including TCH, *β*-cyclocitral, geranylacetone and *β*-ionone. The sum of the five norisoprenoid compound concentrations also showed a significant increase in the *VvGGPPS*-over expressed leaves (**Figure 6C**). The transient over-expression of *VvGGPPS* was also conducted in the leaves of *N. benthamiana* (**Figure 7A**). RT-PCR analysis demonstrated that the *VvGGPPS* was indeed over-expressed in the heterologous transformation system (**Figure 7B**). There were six norisoprenoid compounds were identified in tobacco leaves, of which MHO, *β*-cyclocitral, TDN, and *β*-ionone were significantly increased. Moreover, the sum of the concentrations of the six compounds was significantly increased (**Figure 7C**). The above results indicated that *VvGGPPS* is involved in manipulating the production of norisoprenoids, very likely at the genetic level.

It is known that norisoprenoid biosynthesis in the developing grape berry is mainly completed in the plastids. In order to better illustrate the function of *VvGGPPS*, we carried out an on-line analysis of subcellular localization using the following two websites: http://www.csbio.sjtu.edu.cn/bioinf/Cell-PLoc-2/ and https://rostlab.org/services/loctree2/. Both results showed that the encoded protein is located on the chloroplast. An evolutionary tree was then constructed using the reported plants GGPPS sequences. It was found that VvGGPPS (VIT_205s0020g01240) was clustered in the same clade as the other five protein sequences for which the subcellular localization was experimentally demonstrated (**Supplementary Figure S7**). The target VvGGPPS in this study presented the shortest distance from the rice XP 015647986 and tomato Solyc11g011240 sequences, both of which have been experimentally shown to be in chloroplasts (Zhou et al., 2017; You et al., 2020; Zhou and Pichersky, 2020; Barja et al., 2021). Chloroplasts are a type of plastid. Therefore, it was suggested that VvGGPPS (VIT_205s0020g01240) in this study performed the function in the grapevine plastid.

## 4 Discussion

This study identified the SNP loci and candidate genes genetically controlling norisoprenoids in grape berries using a hybrid population and a germplasm population of grapevine. There have been several reports on the QTLs associated with norisoprenoids in fruits. The first report may be about the QTLs associated with *β*-damascenone in apple, which were found on the LG D-16 using the F1 hybrid population of apple cultivars ‘Discovery’ and ‘Prima’ (Dunemann et al., 2009). Subsequently, the QTLs related to *β*-ionone and *β*-damascenone were also identified in red raspberry (Paterson et al., 2013) and peach fruits (Eduardo et al., 2012). Recently, using a crossing population of *Vitis labruscana* × *Vitis vinifera*, a large common QTL on LG2 was found to be associated with five norisoprenoid compounds in the grape berries. This QTL was found to explain 39.6%-56.1% of the variation in *β*-damascenone, 16.1%-30.8% for *β*-ionone, 16.9%-32.6% for TPB, 21.3%-40.3% for actinidol 1 and 22.2%-40.1% for actinidol 2 (Koyama et al., 2022). This is the first report of the QTLs associated with norisoprenoid compounds in grape berries. However, the previous studies lack further gene exploration and function identification for these QTLs. Our study further investigated the genes corresponding to important SNP loci and elucidated the function of *VvGGPPS*.

### 4.1 Function of *VvGGPPS* in regulating norisoprenoids production

This study identified three SNP loci within *VvGGPPS* (VIT_205s0020g01240) gene that are highly associated with MHO and *β*-cylocitral contents using a hybrid F_1_ population and one germplasm population. Among them, the SNP locus at chr5:2987350 was located in the CDS region of the gene, and the mutation from C to T at this locus, resulting in the substitution of glycine (G) at position 261 with arginine (R), led to an increase in the contents of MHO and *β*-cylocitral, eventually enhancing the total norisoprenoid content.

GGPPS acts at the branch point, moving toward monoterpenoid and norisoprenoid synthesis in the isoprenoid metabolism pathway. This enzyme in plants is responsible for the synthesis of GGPP. In plastids, GGPP is further catalyzed by phytoene synthase (PSY) to produce phytoene, which is then catalyzed by phytoene desaturase (PDS) and other enzymes into carotenoids, which are the precursors of norisoprenoids. Besides, GGPP also serves as a common precursor of tocopherol, abscisic acid, solanum lactone, gibberellin, plastoquinone, and chlorophyll (Kojima et al., 2000). In short, GGPPS is involved in plant growth and development, photosynthesis, signal transduction, environmental adaptation, and abiotic stress (Bouvier et al., 2005; Gershenzon and Dudareva, 2007), and is regarded as a node of several important secondary metabolic pathways in plants. Therefore, it is possible that altering the activity of GGPPS could indirectly manipulate the content of downstream metabolites, including norisoprenoid derivatives.

To date, GGPPS has been intensively studied in a variety of plants, mainly focusing on the function of GGPPS paralogs in particular tissues and cell compartments. It has been demonstrated that *SlGGPPS1* in tomato is responsible for GGPP synthesis in roots, and *SlGGPPS2* and *SlGGPPS3* are in charge of supplying GGPP to produce carotenoids and other isoprenoids in leaves and fruits (Stauder et al., 2018). The lack of *SlGGPPS3* results in the decline of carotenoids and chlorophylls, and thus decreasing the photosynthetic activity of the leaves, whereas both *SIGGPPS2* and *SIGGPPS23* mutants exhibit the defects of pigmentation and ripening in fruits owing to the reduction of GGPP supply (Barja et al., 2021). These results indicate that the GGPPS family are involved multiple biological processes. However, we noticed that previous research regarding the function of GGPPS has mostly focused on the accumulation of carotenoids (Maudinas et al., 1977; Fraser et al., 2000) and chlorophyll (Liang et al., 2002; Ji et al., 2009; Orlova et al., 2009), possibly because they are important pigments in fruits or leaves. Norisoprenoids are the breakdown products of carotenoids; however, the role of GGPPS in the production of norisprenoids has not been well concerned. At present, we firstly report both the genotypes of three SNP loci within *VvGGPPS* (VIT_205s0020g01240), and the gene expression level are highly associated with the content of norisoprenoids (in particular MHO and *β*-cylocitral) in grapes. CCD is a key enzyme that cleaves multiple carotenoids into norisopnoid derivatives. It has been known that lycopene is the precursor of MHO, *ξ*-carotene is the precursor of geranylacetone, and *β*-carotene is the common precursor of *β*-cyclocitral and *β*-ionone (Lashbrooke et al., 2013; Rubio-Moraga et al., 2014). Because both lycopene and *β*-carotene act at the downstream of GGPP in the isoprenoid metabolic pathway, GGPPS is likely involved in the genetic regulation of these norisoprenoid compounds.

There have been some previous examples pointing to that GGPPS is involved in the genetic regulation of certain traits. One study shown that an SNP associated with *p*-cymene in *Eucalyptus grandis* mapped to the gene encoding GGPPS, explaining 3.66% of the phenotypic variation (Mhoswa et al., 2022). However, no further validation was performed in this study. Another study on the the secondary metabolites of *Eucalyptus globulus* leaves also showed that SNP ggpps103 was a non-synonymous mutation SNP in exon 1 of *ggpps*, which has a significant effect on the ratio of monoterpenoids to sesquiterpenoids. At this locus, the homozygous CC allele corresponded to the highest ratio of mono- to sesquiterpenes, whereas the homozygous TT corresponded to the lowest ratio, and the heterozygous TC allele between these two extremes (Kulheim et al., 2011). The authors speculated that this SNP locus could result in the changes in enzyme kinetic of GGPPS, and further change the availability of the substrate pool for *gpps*, thus indirectly influencing the ratio of mono- to sesquiterpenes. This report thus highlighted the potential for the genetic regulation of GGPPS genes on terpenes.

In our study, the SNP locus of *VvGGPPS* at chr5:2987350 was a non-synonymous mutation, leading to the replacement of glycine with arginine. Glycine is a hydrophobic amino acid, whereas arginine is hydrophilic. The alteration of amino acid from hydrophobic to hydrophilic might affect enzyme activity or substrate affinity. Therefore, further biochemical analysis is required.

In addition, other two SNP loci of *VvGGPPS* at chr5:2988230 and chr5:2989379 were also identified to be associated with the contents of MHO and *β*-cyclocitral, respectively, in the hybrid F_1_ population. Overall, the first SNP was localized in the 5′-UTR of the gene, and the mutation from C to G at chr5:2988230 was related to the increase in the content of MHO. The second SNP was located in the intron region of the gene, and the mutation from T to G at chr5:2989379 was related to the accumulation of *β*-cyclocitral. It has been reported that the 5′-UTR of genes plays an important role in determining mRNA stability and translation efficiency. Many studies in different species have revealed that the SNPs in 5′-UTR can also influence phenotypic traits such as the isoflavone content in soybean (Chu et al., 2017), oleic acid content in *Olea europaea* L. (Salimonti et al., 2020), anthocyanin content in the leaves of rice (Khan et al., 2020), *α*-carotene content in maize kernel (Zhou et al., 2012), grain yield in rice under salt stress (Liu et al., 2019) and so on. A GWAS study identified that the SNP located in the 5′-UTR of the *GmMYB29* gene was significantly correlated with the isoflavone content in soybean. Moreover, the analyses of *GmMYB29* expression in the 30 soybean accessions representing varieties with high, medium, and low isoflavone contents, together with the overexpression and RNA interference -mediated silencing of *GmMYB29*, all demonstrated that the *GmMYB29* expression level is highly correlated with the isoflavone content (Chu et al., 2017). Based on these results, it is suggested that the SNP loci in the 5′-UTR could influence the expression levels of genes. Similarly, another study involving three SNPs and one 6-bp deletion located in the 5′-UTR of *OsPL6* gene also indicated that these loci could alter the expression of *OsPL6*. By the combinating of the mapping, sequencing and further marker confirmation, it was demonstrated that the mutations of these SNPs led to the addition of new *cis*-elements in the promoter, which may be the reason for the activation of *OsPL6* gene and the promotion of anthocyanin in leaves (Khan et al., 2020). However, different from the above research results, our data indicate that the genotype differences at chr5:2988230 and chr5:2989379 are not completely associated with the expression of *VvGGPPS* (**Figure 5**). The functions of these two SNP loci remain uncertain.

In summary, the present study suggests that the non-synonymous mutation SNP locus at chr5:2987350 in *VvGGPPS* could be developed as a molecular marker for future using as a potential target for the genetic improvement of norisoprenoids. Future research should be conducted on the transcriptional regulation of *VvGGPPS* since the gene expression level was found to be positively correlated with the accumulation of norisoprenoid derivatives.

### 4.2 Function of *VvDXS1* in regulating norisoprenoids production

In addition to *VvGGPPS*, the two SNP loci at chr5:3851898 and chr5:3854251 within the *VvDXS1* (VIT_205s0020g02130) gene were also identified to be significantly associated with the contents of MHO, *β*-cyclocitral and geranylacetone in this study. DXS is an entrance enzyme and rate-limiting enzyme in the MEP pathway that catalyzes the condensation of the initial substrates, glyceraldehyde-3-phosphate and pyruvate, into 1-deoxy-D-xylulose-5-phosphate (DXP) (Battistini et al., 2016). Five DXS genes have been predicted in the grape genome (Wen et al., 2015). Previous studies have demonstrated that the *VvDXS1* (VIT_205s0020g02130) gene on LG5 is highly associated with the contents of monoterpenes such as linalool, nerol and geraniol in grape berry by means of QTL analysis (Battilana et al., 2009). A gain-of-function mutation from G to T at SNP1822 on *VvDXS1* (VIT_205s0020g02130) results in the substitution of lysine (K) at position 284 with asparagine (N), causing a significant increase in monoterpenoids, and it is proved that this mutation improves the catalytic efficiency and thus changes the enzyme kinetics (Battilana et al., 2011). Interestingly, our study found that the SNP at chr5:3854251 in the coding region of *VvDXS1*, which just corresponds to the SNP 1822 reported previously, is highly associated with the increased accumulation of MHO, *β*-cyclocitral, and geranylacetone. This indicates that the non-synonymous mutation at this locus not only influences the contents of monoterpenes but also those of norisoprenoids. This might be related to the specific function of DXS at the entrance of the MEP pathway. The three compounds, MHO, *β*-cyclocitral, and geranylacetone, are generated from the cleavage of lycopene and carotenoids, which are at the downstream of the MEP pathway. From this perspective, we can infer that the GWAS results of this study are reliable.

In addition, another SNP locus at chr5:3851898 was also identified to be associated with the contents of MHO, *β*-cyclocitral, and geranylacetone in both the hybrid F_1_ population and the gerplasm population. However, the SNP chr5:3851898 is located in the intron region of the gene, and the mutation from A to C at this locus results in the increase of MHO, *β*-cyclocitral and geranylacetone contents. Therefore, the mechanism by which this SNP locus generally regulates the production of norisoprenoid compounds remains unclear at the present, and further research is required in this regard.

Until now, no direct evidence has explained the positive correlation between the expression of *DXS* and the contents of norisoprenoid derivatives. The previous research has almost concentrated on the effect of the concentration of carotenoids, rather than norisoprenoids (Lois et al., 2000; Estevez et al., 2001; Munoz-Bertomeu et al., 2006; Paetzold et al., 2010; Nieuwenhuizen et al., 2015). The coordinated expression of *DXS* with genes of the downstream pathway has been reported in some transgenic plants. For example, in the transgenic *Arabidopsis thaliana* overexpressing potato *DXS*, both *GGPPS* and *PSY* were upregulated, consequently promoting the increase of lutein and *β*-carotene (Henriquez et al., 2016; Simpson et al., 2016). In this study, we investigated the gene expression in the F_1_ individuals with extremely high and low contents of the associated components, and found that *VvDXS1* expression, like that of *VvGGPPS*, completely follow a similar trend as the components content.

DXS as an entrance enzyme of the MEP pathway, should not function alone. Some researchers have found that when kiwifruit *DXS* and *TPS* genes were simultaneously transferred into tobacco, the content of terpene was increased by 100-fold, which was much more efficient than transferring one of the genes alone (Nieuwenhuizen et al., 2015). In grapes, the combined overexpression of *VvDXS1* and *VvTPS56* significantly improved linalool and geraniol in the leaves of *N. benthamiana* (Wang et al., 2021). A recent study also demonstrated that simultaneous overexpression of *VvDXS4* and *VvTPS59* in the transient transgenic system using *V. quinquangularis* leaves had better promotion to monoterpenes, including linalool, linalool oxide, geraniol, citronellol, and trans-rose oxide, in comparison with the overexpression of *VvTPS59* alone (Liu et al., 2022). These results indicate that the increased *DXS* expression can drive the carbon flow of the isoprenoid pathway to the downstream monoterpene biosynthesis. In this study, we failed to achieve simultaneous overexpression of *VvDXS1* and *VvGGPPS* in the transient systems using *V. quinquangularis* and tobacco leaves. However, from the available data, we can conclude that the co-expression of these two genes manipulates the synthesis of norisoprenoid compounds.

### 4.3 Potential role of other candidate genes

Based on GWAS of the hybrid population and KASP of the germplasm population, the SNP loci within carotene *ϵ*-monooxygenase (*LUT1*) and carbohydrate kinase genes were also shown to be associated with the contents of norisoprenoids. Clearly, the proteins encoded by these genes are not directly involved in the norisoprenoid biosynthetic pathway. Meanwhile, the SNP loci within some genes coding for transcription factors were also identified to be related to the content of norisoprenoid compounds, including squamosa promoter-binding-like protein 1 (SPL), BSD domain-containing protein, zinc finger protein (ZFP), bZIP protein HY5, and nuclear transcription factor Y subunit B-5. There were a total of ten significant SNP loci mapped to these above seven genes. The genetic control of these SNP loci on the levels of norisoprenoids remains unclear according to the present study.

Currently, some transcription factors have been reported to be involved in the genetic regulation of certain traits. For example, squamosa promoter-binding-like protein 1 is a kind of transcription factor widely existing in green plants. Studies have shown that SPL is involved in the genetic regulation of flowering time in C_4_ rhizomatous grasses *Miscanthus* and switchgrass (Jensen et al., 2021), and in the genetic controlling of resistance to angular leaf spot in common bean (Oblessuc et al., 2013). In our study, three SNP loci were located within the transcription factor SPL, among which the locus at chr5:2565231 was in the 3′-UTR of the gene, whereas the loci at chr5:2566542 and chr5:2565775 were in the CDS region of the gene. The mutations at these SNP loci increase the concentration of MHO and *β*-cyclocitral. At present, there are few studies on the BSD domain-containing protein in plants. QTL studies on the responses towards salinity stress of rice seedlings showed that the expression of some genes encoding ‘transcription factors’, including *OsBSD* in *Saltol* QTL (SalTFs), could play an important role in the salinity stress response, directly or indirectly (Nutan et al., 2017). In this study, one SNP at chr5:4112382 was located within the CDS region of the BSD domain-containing protein gene, and the mutation at this locus caused the variation in the amino acid sequence, leading to an increased content of MHO, suggesting that this protein may be involved in the regulation of MHO synthesis.

Zinc finger proteins are a common protein family in eukaryotes that performs a wide range of biological functions. Previous studies primarily indicate that these proteins are involved in the genetic regulation of ear height in maize; spike morphological traits, plant height, and heading date in wheat; and chloroplast development and immature pepper fruit color in *Capsicum chinense* (Li et al., 2016; Borovsky et al., 2019; Xu et al., 2022). In this study, two SNP loci at chr5:3820289 and chr5:3826510 were located within the 3′-UTR and intron region, respectively, of the gene. The mutations at the two SNP loci increased the concentrations of MHO and geranylacetone. Regarding bZIP transcription factor, it has been reported to participate in the genetic regulation of resistance to high-intensity ultraviolet-B stress in soybean leaves, which explained 20% of phenotypic variation (Yoon et al., 2019). In this study, one SNP at chr5:2861511 was located within the 3′-UTR of the bZIP protein HY5, and the mutation at this position increased the content of MHO. In general, previous studies on the genetic regulation of these transcription factors have mainly focused on the growth and development, and resistance-related traits in plants. However, there have been no relevant reports involving the genetic regulation of aromatic compounds such as norisoprenoids. The present study merely provided the possibility. Further researches should be needed to make clear whether these transcription factors are involved in the regulation of norisoprenoid biosynthesis in grapes.

In summary, based on the genome-wide association analysis of a hybrid F_1_ population and KASP verification of a grapevine germplasm population, we identified 13 SNP loci that were significantly associated with norisoprenoids contents in the grape berry, including two non-synonymous mutations SNPs within the *VvDXS* and *VvGGPPS* genes in the isoprenoid pathway. Genotype analysis revealed that the grapevine individuals with the heterozygous genotype C/T at chr5:2987350 of *VvGGPPS* could accumulate higher average levels of 6-methyl-5-hepten-2-one and *β*-cyclocitral than those with the homozygous genotype C/C. The transient overexpression of *VvGGPPS* in the leaves of *Vitis quinquangularis* and tobacco, respectively, indeed increased the content of norisoprenoids. These findings provide a potential marker for improving “floral/fruity” aromas characterized by norisoprenoids through molecular breeding. Meanwhile, this work also lays a foundation for dissecting the regulation of norisoprenoids accumulation, a group of important aroma compounds in wine grape berries. In the future, we will continue to explore the genetic regulation of other SNP loci and the candidate genes identified in this study.

## Data availability statement

The genome re-sequencing datasets supporting the results of this article can be found in the (China National Center for Bioinformation National Genomics Data Center) CNCB-NGDC (https://ngdc.cncb.ac.cn/) under accession number CRA009145. Other data supporting the findings of this study are available within the paper or its supplementary data.

## Author contributions

QS and LH performed the research, data analysis and original draft preparation. QS completed the manuscript writing and editing. LS and H-YX provided hybrid F_1_ population. Z-YS and Y-QF performed the part of chemical analysis. Q-HP supervised and guided the research. Q-HP and C-QD provided the funding. All authors contributed to the article and approved the submitted version.
