# Supplementary figures and images for: Identification of SNP loci and candidate genes genetically controlling norisoprenoids in grape berry based on genome-wide association study
Source: Front Plant Sci. 2023 Mar 1;14:1142139. doi: 10.3389/fpls.2023.1142139 (PMC10014734; doi:10.3389/fpls.2023.1142139)

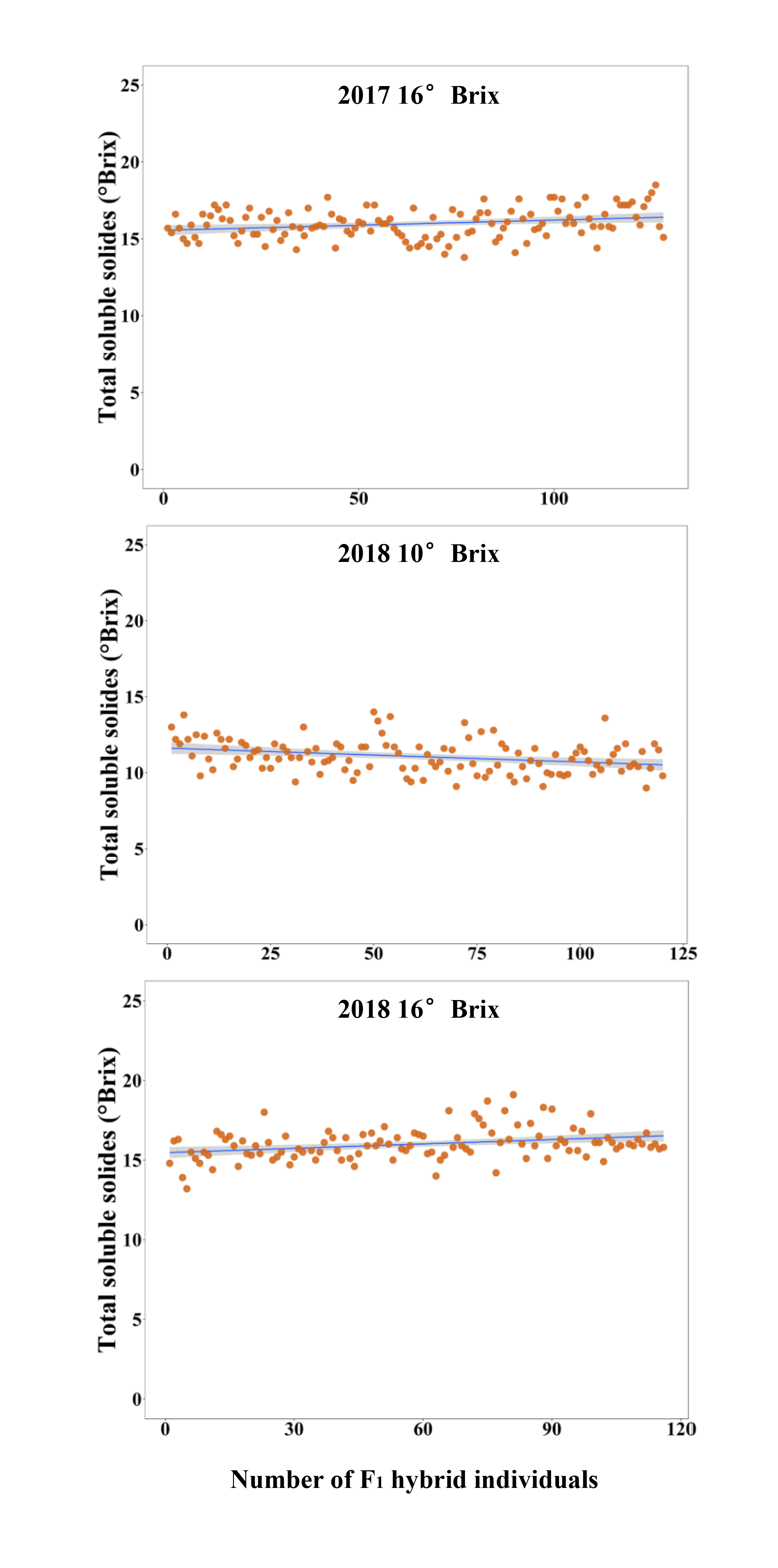

Supplement: Supplementary file 1 [file Image_1.tif]

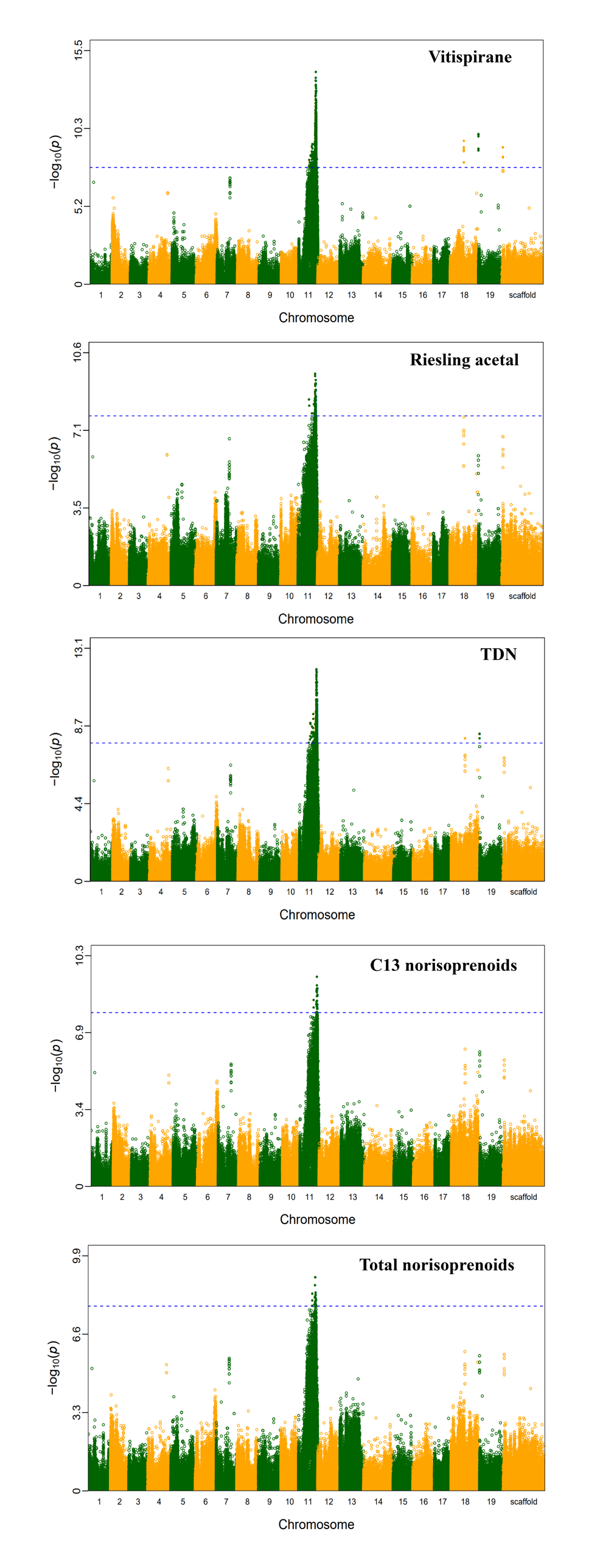

Supplement: Supplementary file 2 [file Image_2.tif]

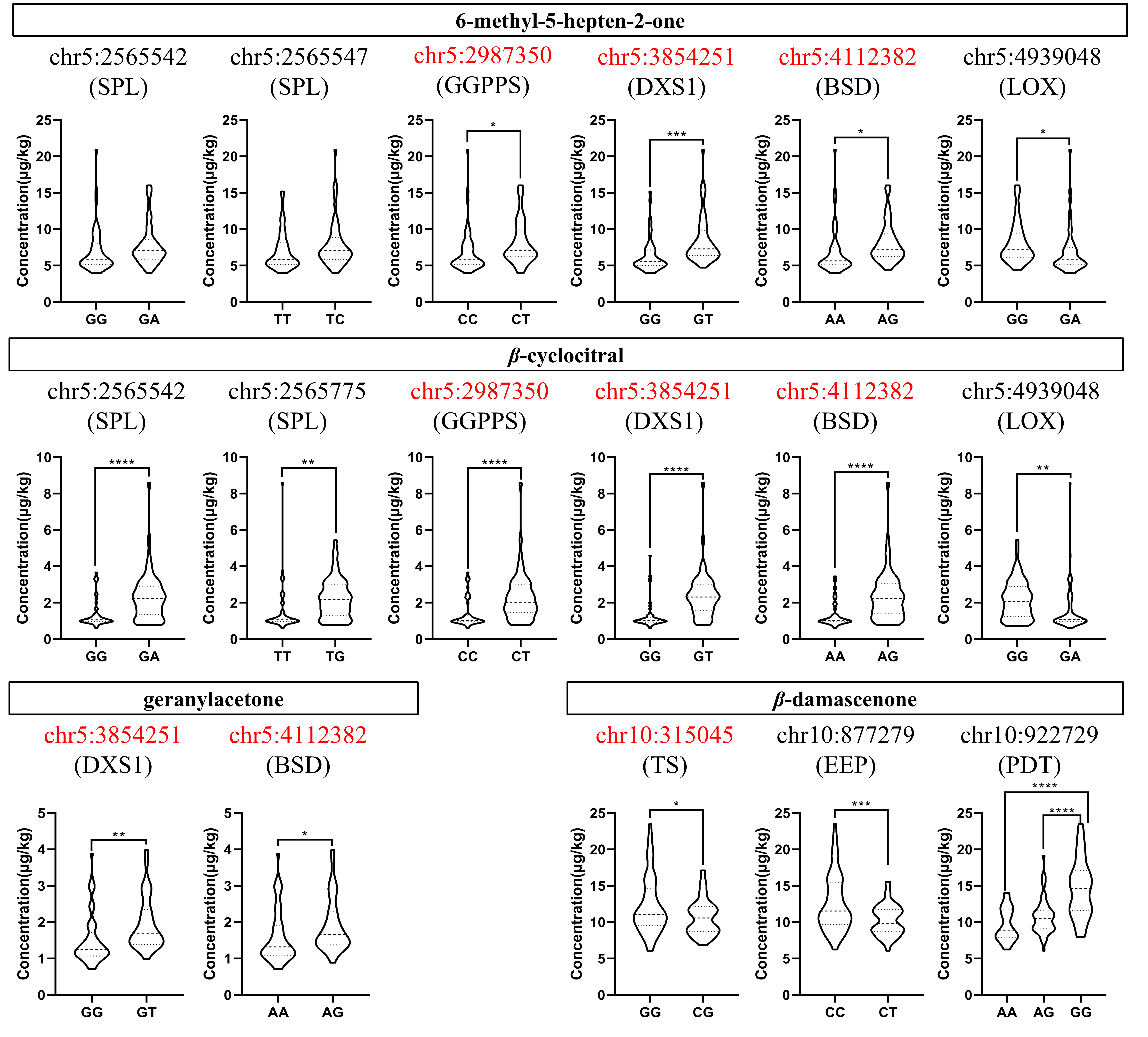

Supplement: Supplementary file 3 [file Image_3.tif]

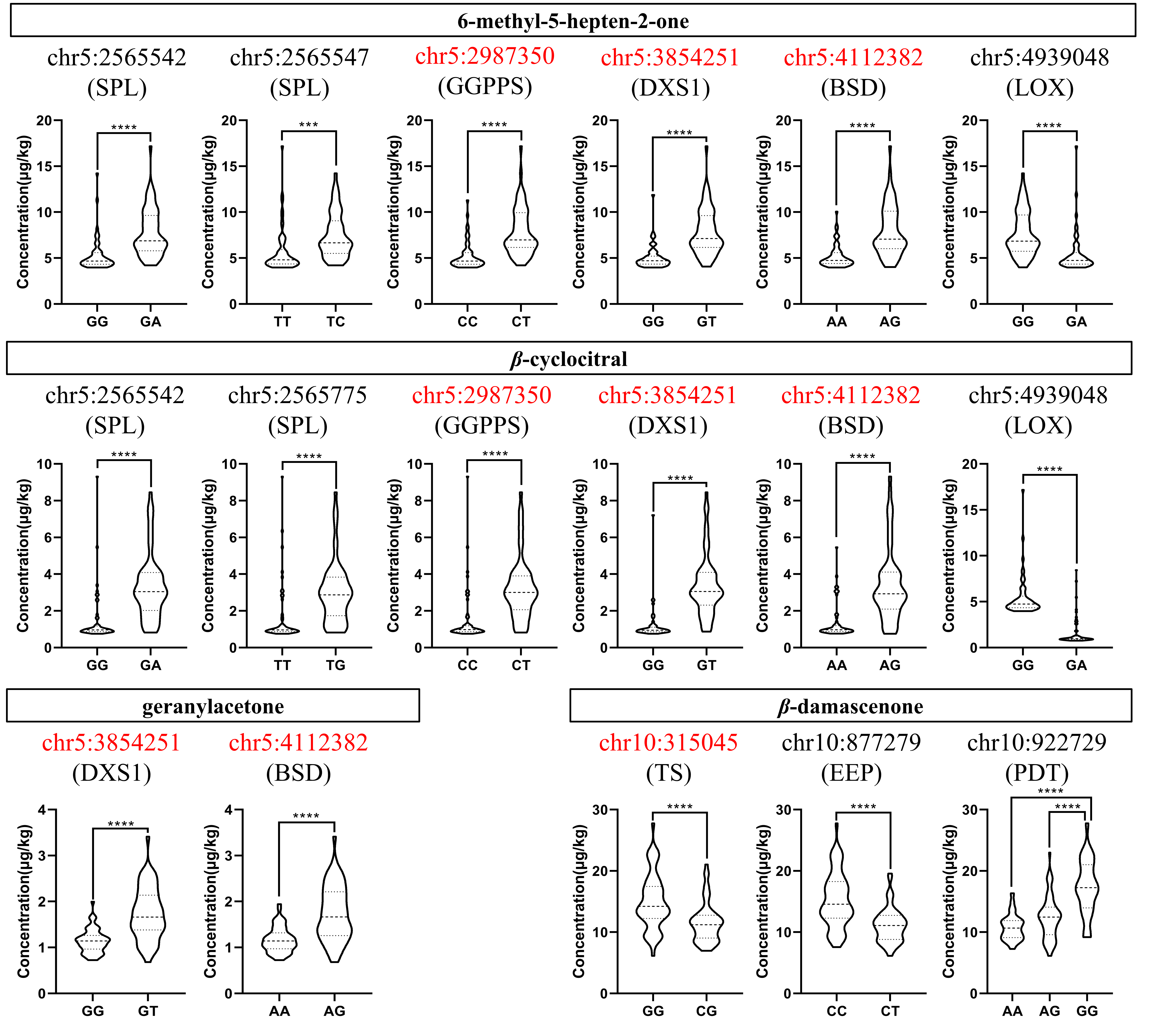

Supplement: Supplementary file 4 [file Image_4.tif]

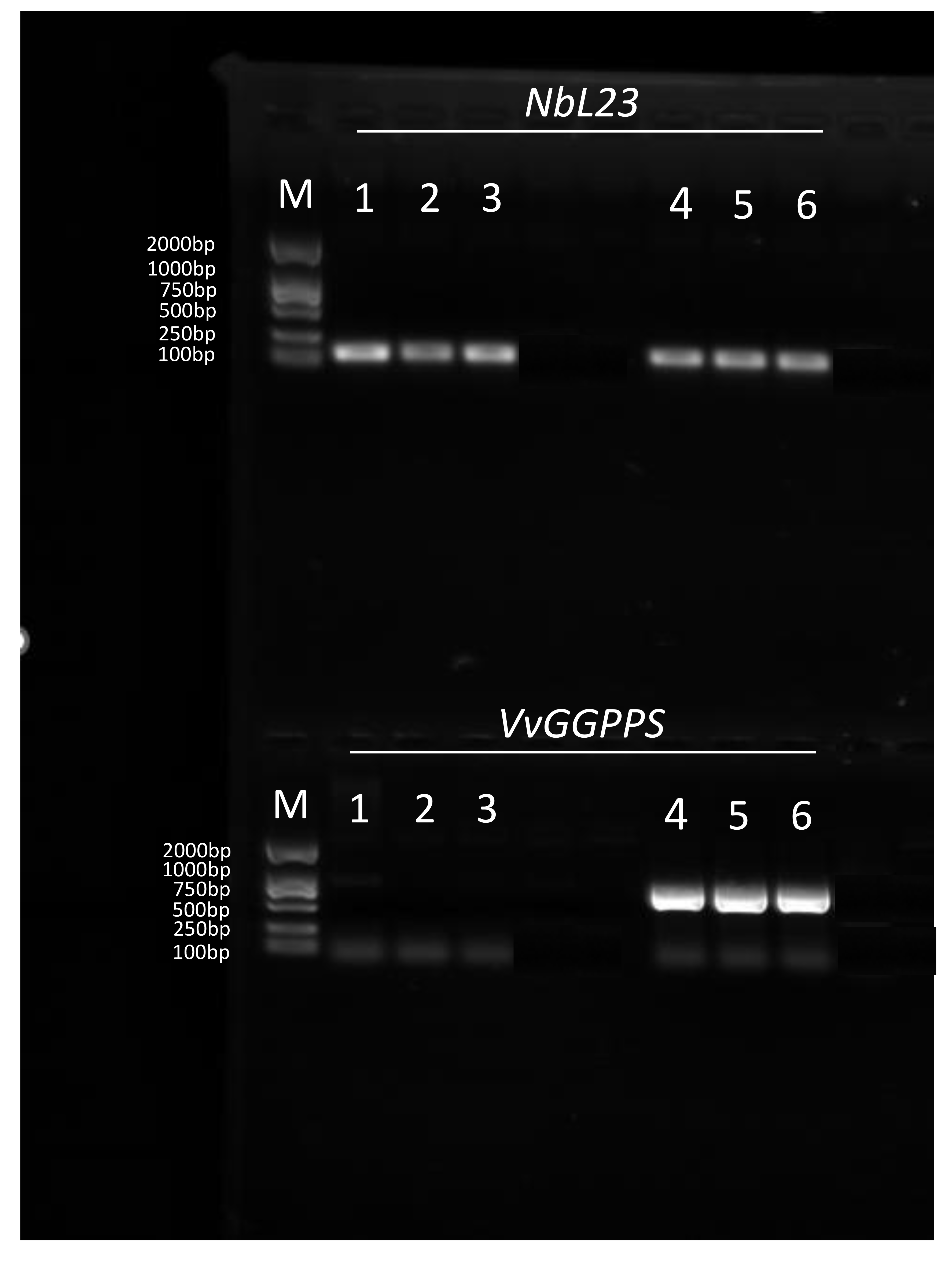

Supplement: Supplementary file 5 [file Image_5.tif]

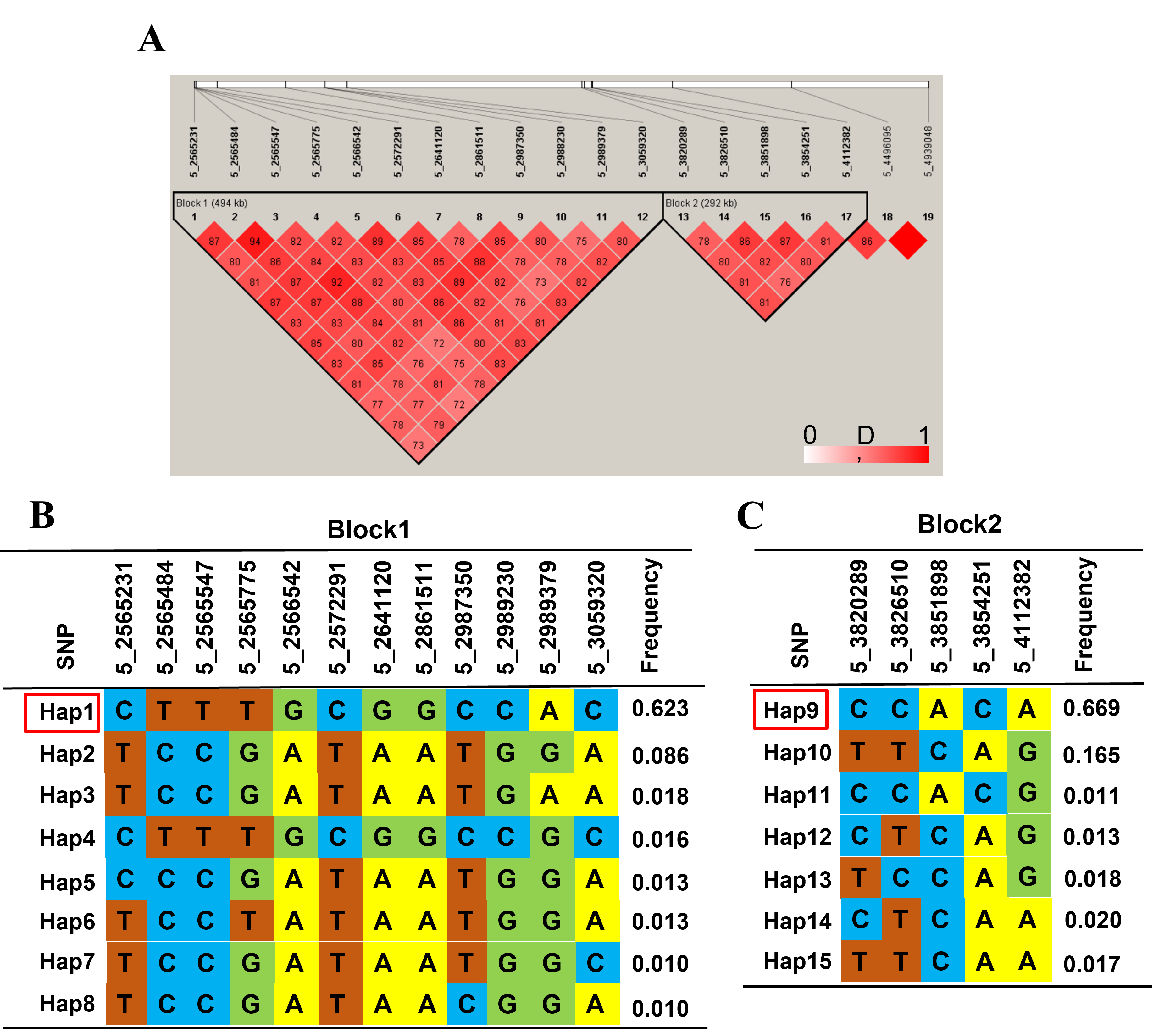

Supplement: Supplementary file 6 [file Image_6.tif]

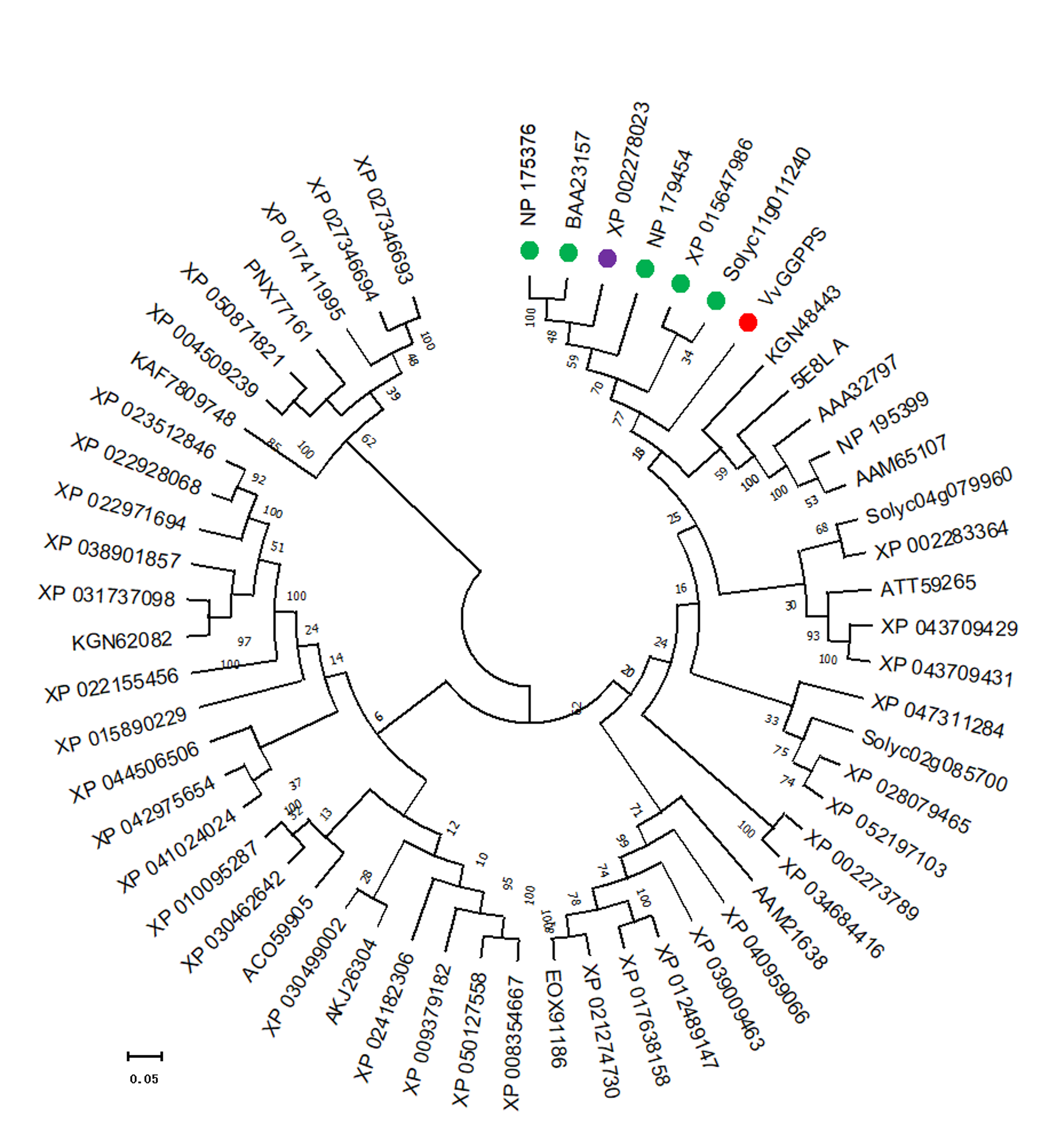

Supplement: Supplementary file 7 [file Image_7.tif]
